# Molecular dynamics simulations reveal ligand-controlled positioning of a peripheral protein complex in membranes

**DOI:** 10.1038/s41467-016-0015-8

**Published:** 2017-02-23

**Authors:** Steven M. Ryckbosch, Paul A. Wender, Vijay S. Pande

**Affiliations:** Departments of Chemistry and of Chemical & Systems Biology, Stanford University, Stanford, California 94305 USA

## Abstract

Bryostatin is in clinical trials for Alzheimer’s disease, cancer, and HIV/AIDS eradication. It binds to protein kinase C competitively with diacylglycerol, the endogenous protein kinase C regulator, and plant-derived phorbol esters, but each ligand induces different activities. Determination of the structural origin for these differing activities by X-ray analysis has not succeeded due to difficulties in co-crystallizing protein kinase C with relevant ligands. More importantly, static, crystal-lattice bound complexes do not address the influence of the membrane on the structure and dynamics of membrane-associated proteins. To address this general problem, we performed long-timescale (400–500 µs aggregate) all-atom molecular dynamics simulations of protein kinase C–ligand–membrane complexes and observed that different protein kinase C activators differentially position the complex in the membrane due in part to their differing interactions with waters at the membrane inner leaf. These new findings enable new strategies for the design of simpler, more effective protein kinase C analogs and could also prove relevant to other peripheral protein complexes.

## Abstract (web summary)

Natural supplies of bryostatin, a compound in clinical trials for Alzheimer’s disease, cancer, and HIV, are scarce. Here, the authors perform molecular dynamics simulations to understand how bryostatin interacts with membrane-bound protein kinase C, offering insights for the design of bryostatin analogs.

## Introduction

A major challenge in chemistry and structural biology is the determination of the structure and dynamic function of membrane-associated proteins and their regulatory ligands in their membrane environment. Representative of such peripheral proteins, protein kinase C (PKC) isoforms are of exceptional current interest due to their proposed role in major unmet medical needs, including the eradication of HIV/AIDS^1–3^, treatment of Alzheimer’s disease^4^, and small molecule-enhanced cancer immunotherapy^5^. The activities of the conventional and novel subfamilies of PKC are modulated endogenously by the binding of diacylglycerol (DAG) to the highly homologous PKC C1a and C1b regulatory domains. A variety of natural products, including plant-derived phorbol esters^6^ and prostratin and marine-derived bryostatin 1 (henceforth bryostatin), compete with mammalian DAG for binding to PKC’s C1 domains, but are more potent and elicit different biological activities from DAG and each other. The phorbol esters, for example, are potent tumor promoters, while the structurally related prostratin, a C12-deoxyphorbol-13-acetate, is not. It is, however, a lead candidate in efforts to eradicate HIV/AIDS^7^. Bryostatin, a marine macrolide reported by Pettit and coworkers in 1982 (ref. ^8^), is not tumor promoting, but instead is a highly promising therapeutic lead due to its immunomodulatory activity^5^, and is currently in clinical trials for the treatment of Alzheimer’s disease^9,10^ and has recently completed a clinical trial for the activation of latent HIV reservoirs as part of an HIV eradication strategy^11^. Bryostatin is also of clinical interest in connection with the treatment of fragile X syndrome^12^, Niemann-Pick disease^13^, and Charcot-Marie-Tooth disease^14^.

Notwithstanding the proposed role of different PKC isoforms in these diseases, the affinities and selectivities of PKC ligands do not adequately explain their differing activities, suggesting that activity could be a function of other factors, such as the location and membrane environment of the PKC–ligand complexes. Pertinent to this point, the structure and dynamics of PKC C1b–ligand complexes in a membrane environment are not known. This is especially significant because the membrane environment has been shown to dramatically influence ligand binding. For example, the affinities of PKC modulators to the regulatory C1b domain are strongest in the presence of a vesicle consisting of phosphatidylserine (PS)^15,16^, while in the absence of PS, PKC binding is drastically reduced. Similarly, cytosolic PKC is inactive, while the membrane-associated PKC–ligand complex is active^17,18^. Structural information pertinent to this membrane complex is thus a required starting point for understanding, at the molecular level, how different PKC activators affect the membrane positioning of PKC and thus its interaction with client proteins. This would further provide the necessary structural information for designing new ligands to selectively regulate these interactions, a largely under-explored goal. Traditionally, X-ray crystallography or solution nuclear magnetic resonance (NMR) have been employed to access structures of protein–ligand complexes, and these techniques have contributed to our understanding of PKC’s static crystal lattice and solution structures. However, these approaches are of only limited usefulness for ligand-bound, membrane-associated structures. Indeed, only one X-ray structure of a PKC C1b domain with a bound weak activator is known^19^. Co-crystallization problems have precluded studies on more relevant ligands, including bryostatin and prostratin. Moreover, this structure lacks the PS membrane, which has been shown to be critical to PKC binding and activity^15^. More generally, such an approach entrenches the view that there is but one relevant bound structure, while it has been shown that activation of peripheral membrane proteins is complex and often results in many relevant states. While limited studies have appeared on the design of PKC modulators over the last 30 years, none has been based on multi-state dynamic structures in a membrane environment.

Previously, we reported the first designed PKC modulators based on computer-guided comparison of pharmacophoric features of naturally occurring PKC ligands (bryostatin, phorbol esters, DAG, ingenol, gnidimacrin, and teleocidin)^20–22^. While these ligand comparisons have identified common pharmacophores that could contact the protein, they have not included the protein-binding domain itself. Thus, they do not address how exposed surfaces of the bound ligand might influence the depth, orientation, structure, and dynamics of the PKC–ligand complex in the membrane, issues of fundamental importance to understanding peripheral proteins. Furthermore, they do not an allow investigation of the possible existence of multiple protein states, which is important for peripheral membrane proteins as activation is often a complex, multi-state process^23^. We are now addressing this problem through complementary experimental and computational approaches, the former via solid-state REDOR NMR experiments providing intramolecular distances of labels in a ligand within the PKC C1b–ligand–membrane complex^24^. The latter, described here, provides a uniquely long timescale molecular dynamics (MD) analysis of the structure and dynamics of the PKC C1b–ligand complex in a membrane environment, revealing previously unrecognized aspects of its membrane-bound states.

MD simulations are uniquely suited for examining the structures of membrane-associated protein–ligand complexes with atomistic detail. Although X-ray or solution NMR structures are important starting points for these studies, they do not address the dynamic interactions between the protein–ligand complex and the lipids and waters influencing membrane association, or the influence of multiple protein–ligand states. In the MD simulations described below, we resolve an experimentally inaccessible structure using Markov State Models (MSMs) over long timescales (400–500 µs per system, three orders of magnitude longer than all previous PKC studies)^25–27^, allowing for a comprehensive understanding of the structure and dynamics of the PKC–ligand–membrane complex. We find that different ligands differentially control positioning of the complex in the membrane. This difference results from bryostatin’s previously unaddressed interaction with waters and lipids in the membrane headgroup region, and the conformation this imposes upon the PKC C1b–ligand complex. An enormous and impressive body of knowledge focused on bryostatin alone has been generated over the past 35 years^28^. Due to scarcity, cost, and environmental issues, bryostatin’s natural supply is uncertain^29^ and, as is found for most natural products, bryostatin is not optimized for human use^30,31^. This information provides structural hypotheses required for the design and synthesis of new, simplified, and potentially superior bryostatin analogs, intensely sought after agents for research and clinical studies.

## Results

### Ligands alter favored states of PKCδ C1b domain in membrane

**Fig. 1:**
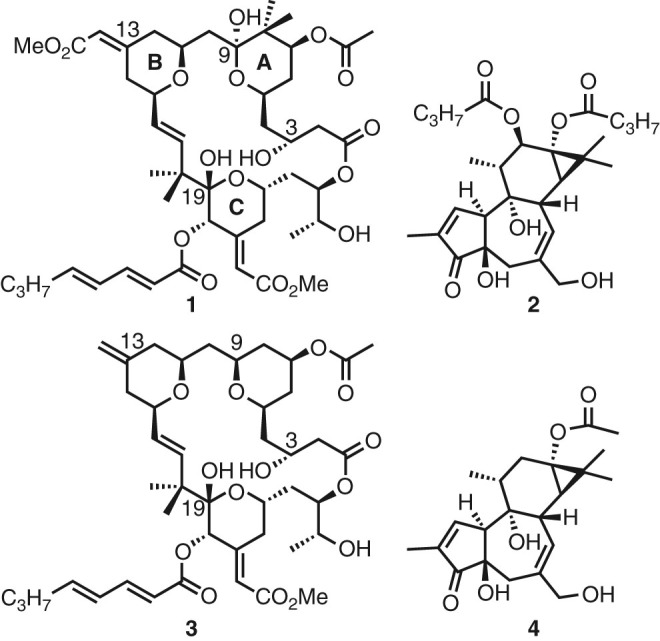
**Structures of compounds simulated.** Clockwise from *top left*: bryostatin (**1**), PDBu (**2**), prostratin (**4**), and a bryolog (**3**). Atom numberings of bryostatin are labeled, as well as bryostatin’s A, B, and C rings. Bryostatin is in the clinic for the treatment of Alzheimer’s disease^9^ and for HIV/AIDS eradication^11^. Prostratin shows PKC selectivities similar to bryostatin (albeit with lower affinities) and is a preclinical lead for HIV eradication. PDBu is similar in structure to prostratin, but unlike it is representative of the tumor-promoting phorbol diesters. Bryolog **3**, synthesized by Keck and coworkers, is structurally similar to bryostatin itself but has been shown to behave quite differently in biological assays

Simulations of the PKCδ C1b domain with each one of four bound ligands (Fig. 1: bryostatin (**1**), phorbol 12,13-dibutyrate (PDBu, **2**), a bryostatin analog (**3**)^32^, and prostratin (**4**)) as well as without a ligand have been performed in the presence of a PS membrane. While these ligands bind competitively and with comparably high affinity, they elicit different biological activities, suggesting that there is more to function than reflected in the PKC–ligand complex alone. The C1b domain is the primary region of interest for probing the impact of PKC activators, and binding affinities of these ligands to lone C1b domains have been shown to be comparable to those with the full-length protein^33^.

**Fig. 2:**
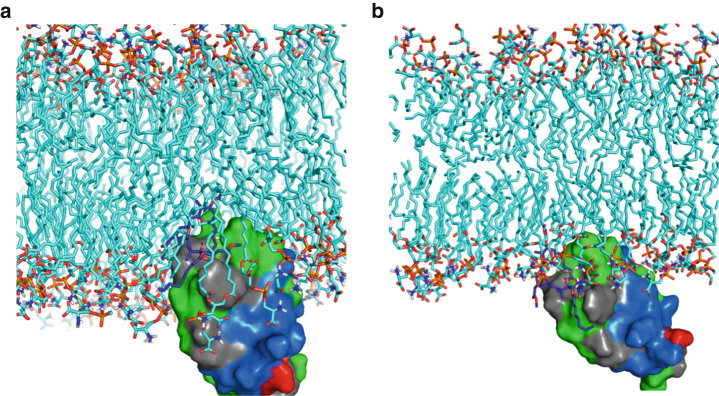
**Representative snapshots of PKCδ C1b–bryostatin–membrane complex.** *Green*, *blue*, and *red* surfaces correspond to hydrophobic, basic, and acidic residues, respectively. In the more deeply embedded structure **a**, many hydrophobic residues are able to reside in the hydrocarbon region of the membrane, while the vertical orientation allows the cationic residues along the side of the protein to interact with the anionic headgroups. In the shallower orientation **b**, fewer hydrophobic residues reside in the membrane, but more cationic residues are able to interact with the anionic headgroups. Such snapshots are similar for the PDBu and ligand-free systems

These protein–ligand complexes were simulated in various membrane orientations; example snapshots are shown in Fig. 2. Analysis of these simulations with MSMs revealed that although all systems were characterized by a free energy minimum when embedded in the membrane, their free energy landscapes varied with different ligands as a function of orientation in the membrane.

**Fig. 3:**
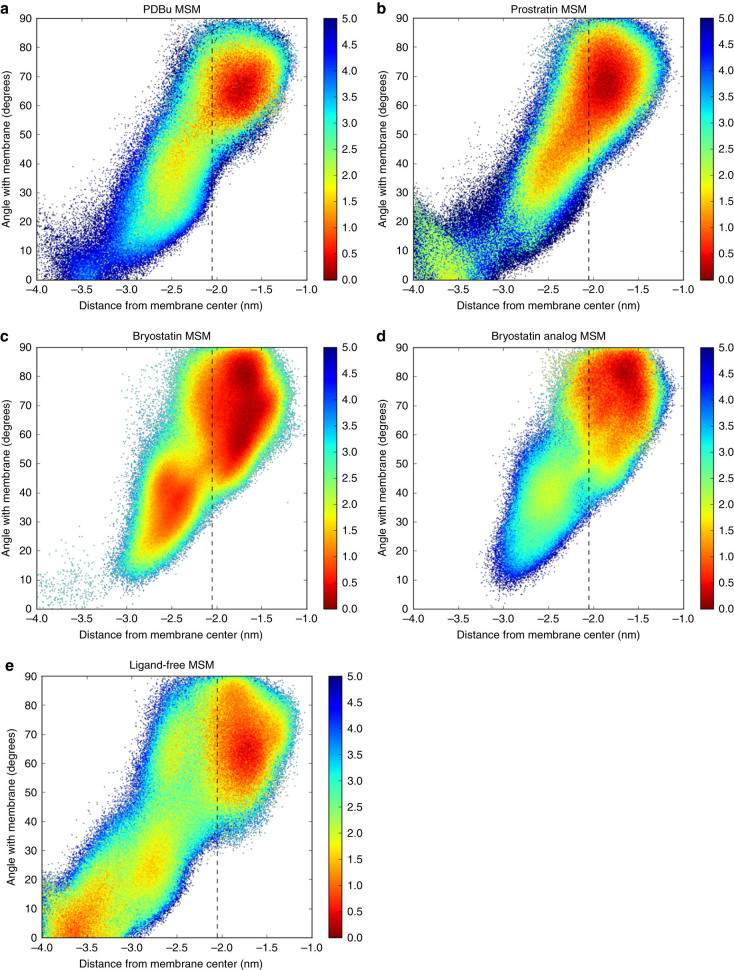
**Free energy as a function of depth and angle of peptide in membrane.** The panels represent the free energies of the peptide in the presence of various ligands (**a**–**d**) and without a ligand (**e**), reported in kcal mol^−1^. Note that bryostatin **c** and (to a lesser extent) prostratin **b** possess two low-free energy wells, one shallow and one deep, while PDBu **a** and bryolog **3** **d** only show one free energy minimum, deeply embedded in the membrane. The *dashed line* indicates the pseudo plane of phosphorus atoms in the bottom leaflet, and distance 0 is the plane through the center of the membrane. Distance is measured from an average of the positions of the alpha carbons of M9, T12, L21, and V25, all atoms near the binding site. The angle is found by first creating a line through this point and an average of the positions of the alpha carbons of F3, G35, N48, and the zinc ion coordinated by H1, C31, C34, and C50, and calculating the angle of this line with the membrane plane. This line goes approximately through the middle of the relatively cylindrical peptide. The range of the 95% confidence interval as determined by bootstrapping simulations was less than 0.1 kcal mol^−1^ for all configurations and all ligands. See Supplementary Fig. 4 and the Supplementary Discussion for further discussion of error bars for these free energies

As seen in the free energy histograms in Fig. 3, in the case of PKCδ C1b complexed with PDBu or bryolog **3**, there is one strongly favored free energy minimum with complexes deeply embedded in the membrane. This configuration embeds the hydrophobic residues near the binding pocket in the hydrophobic core of the membrane, while still allowing for many contacts between the basic residues and the negatively charged PS headgroups. This result is in strong agreement with both experimental^16,34^ and theoretical^26,35,36^ studies of the PS-associated PKC C1b–PDBu complex (see Supplementary Figs. 1–3, and Supplementary Discussion). In contrast, in the case of the PKCδ C1b complexed with bryostatin, and to a lesser extent, prostratin, our MD simulations reveal a second highly stabilized state in a shallower, more angled orientation that is far more favorable than for other ligands. The results for the ligand-free system is similar to those of the PDBu system in that its primary free energy minimum is also deeply embedded, but it is also characterized by a broader free energy landscape, as would be expected from the lack of a ligand to stabilize the membrane-bound complex.

### Waters mediate orientation of C1b–ligand–membrane complex

**Fig. 4:**
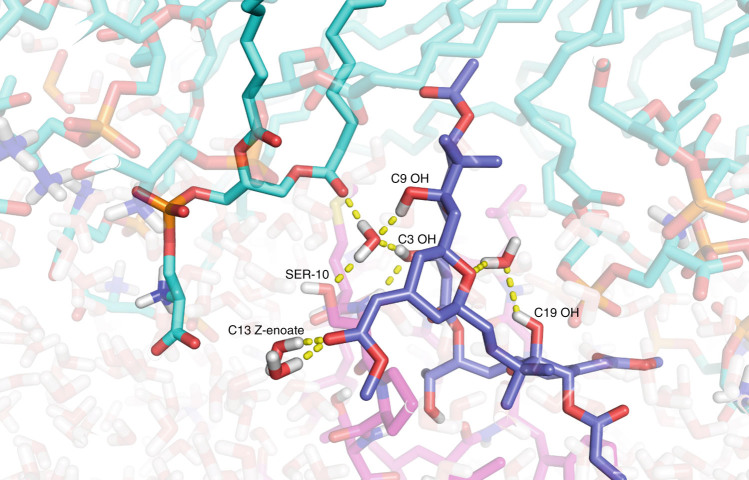
**Structured and unstructured waters coordinating bryostatin in the shallow binding mode.** Waters are highlighted for clarity. Note the coordination of a water with C9 and C3 OH groups, as well as SER10 side chain and a lipid carbonyl oxygen. The C3 OH is also hydrogen bonding with the SER10 backbone amide proton. A structured water also coordinates with C19 OH. Unstructured waters from bulk solvent hydrogen bond as well with the C13 Z-enoate. Solvation of both of these moieties provides stabilization of shallow orientation and a barrier to insertion more deeply into the membrane. Other PKC-binding ligands lack such water-coordinating moieties, and therefore only favor the deeply inserted state. Conversely, altering substitution at deeply embedded C7 and C8 can substantially abrogate binding activity altogether (see Supplementary Fig. 5). The conformation shown of bryostatin is the predominant one of the simulations; see Supplementary Figs. 6 and 7 and Supplementary Discussion for more discussion of bryostatin’s conformations

Although all complexes showed states deeply inserted into the membrane, a striking difference between them is the presence of two thermodynamic basins with regard to the depth and angle of penetration of the bryostatin–PKCδ C1b complex. While the ligand-free and PDBu systems have one dominant orientation deeply embedded in the membrane, bryostatin, in addition to this state, has a favorable shallow and more angled state (see Fig. 2 for snapshots of both the shallow and deep states). This shallow orientation is stabilized in part by strong associations of bryostatin with waters at the membrane interface. In the majority of snapshots in this state, there are several structured waters in the partially hydrated headgroup region of the membrane that hydrogen bond with bryostatin, including one shared between the C3 and C9 hydroxyls and one coordinated by the C19 hemiketal (Fig. 4). These waters reside in the partially hydrated region of the membrane near the lipid carbonyl groups. Furthermore, the B-ring Z-enoate carbonyl resides at the fully solvated water-headgroup interface, where it hydrogen bonds with two bulk waters.

These waters offer significant energetic stabilization to the PKC C1b–ligand complex in this shallow, angled orientation, but are lost as the PKC C1b–ligand complex moves deeper into the membrane. This deep state represents a second energy well due to the embedding of the hydrophobic residues of the peptide into the hydrocarbon region of the membrane. Lacking a similar array of hydrogen bond donors and acceptors, PDBu and ligand-free systems show only one favorable state deeply embedded in the membrane. This analysis provides a new structural hypothesis about the PKC–bryostatin complex that would not be accessible by X-ray, NMR, or ligand-only analyses. Prostratin possesses a free energy landscape similar to both bryostatin and PDBu, and features a substantial but less well-defined shallow state. This can be attributed to the fact that while prostratin is structurally similar to PDBu and lacks bryostatin’s hydrogen-bonding moieties in its northern region, it is also substantially less lipophilic than PDBu^37^. This decrease in surface lipid character disfavors a deeply embedded state and results in a more favorable orientation of the complex that is shallowly associated with the membrane, suggesting that it would have a more bryostatin-like membrane association. It is noteworthy that prostratin is not a tumor promoter and induces similar activities to bryostatin.

To further explore whether this second, shallow free energy basin for bryostatin is the result of coordination of waters by bryostatin’s AB ring region, simulations were performed on bryostatin analog **3**, which lacks the water-coordinating C9 OH and B-ring Z-enoate moieties^32^. For this analog, only the deeply embedded orientation is favored, rather than both the shallow and deep states for bryostatin, suggesting that this bryostatin analog would have more phorbol ester-like association. Whereas the ΔG between the deep and shallow orientations of bryostatin was approximately 0.9 kcal mol^−1^, **3** showed a ΔG of 1.9 kcal mol^−1^ (see Supplementary Methods for calculations). This ΔΔG of 1.0 kcal mol^−1^ is highly significant (see Supplementary Fig. 4 and Supplementary Discussion for discussion of error bars for each system), and suggests that coordination of water by the PKC C1b-ligand system could impact its free energy landscape and affect its positioning on or in the membrane.

### Membrane waters explain known activities of PKC modulators

In 1988, we reported the first computationally derived pharmacophore model for bryostatin’s binding to PKC that provided the basis for the first designed bryostatin analogs, exhibiting affinities comparable or superior to bryostatin itself^21,22^. Guided by this model, much work has been done to simplify bryostatin’s A-ring and B-ring in order to reduce its complexity and produce analogs with bryostatin-like affinity^29,38^. However, these studies focused principally on the PKC C1b-ligand contacts and did not address the exposed surfaces of the ligands that could affect translocation and membrane association. Furthermore, it is known that while bryostatin’s northern region does not directly alter binding affinity, it does play a large role in activities resulting from PKC binding. The Wender and Zack groups have observed, for example, that bryostatin analogs that bear both the C13 Z-enoate or the C9 OH (**5**), or possess only one of these functionalities (**6**, **7**), bear activities similar to bryostatin itself in their J-Lat cell line model of HIV latency activation (see Table 1). Analogs that lack both of these functionalities (**8**, **9**), however, exhibit abrogated activity by as much as two orders of magnitude, even though they all exhibit virtually identical PKC-binding affinity^2^. Keck and coworkers have observed similar trends in their U937 assay for “bryostatin-like” activity (see Table 2). In these studies, analogs lacking both the C13 Z-enoate and C9 OH produce a phorbol ester-like response (**3**)^32,39^, while those retaining either of the two functionalities (**10**, **11**) exhibit bryostatin-like activity^40,41^. This is aligned with the results described above, as the simulated free energy landscape of **3** closely resembles that of PDBu rather than the two-basin landscape of bryostatin (compared in Fig. 3).

**Table 1:** J-Lat activity depends on the northern region of bryostatin, while binding affinity does not^2^


	X	Y	Z	PKC *K*_i_ (nM)	J-Lat EC_50_ (nM)
**5**	CO_2_Me	OH	OAc	0.79	0.38
**6**	H	OH	OAc	0.95	0.46
**7**	CO_2_Me	H	OAc	0.32	1.15
**8**	H	H	H	0.58	37.4
**9**	H	H	OAc	0.42	32.0

Note that the presence of one or both of the C13 Z-enoate or the C9 OH (moieties responsible for the shallow orientation of PKC in the membrane) retains J-Lat activity (as in **5**, **6**, and **7**), but in the absence of both (as in **8** and **9**) activity is significantly lessened. PKC-binding affinity stays constant throughout

**Table 2:** Bryostatin-like activity depends on northern region, while binding affinity does not^29,39,40^


	X	Y	Z	PKCα *K*_i_ (nM)	U937 activity
Bryostatin	CO_2_Me	OH	Me	1.35	Bryostatin
**10**	H	OH	Me	0.52	Bryostatin-like
**11**	CO_2_Me	H	Me	0.38	Bryostatin-like
**3**	H	H	H	3.0	Phorbol ester-like

In the presence of either the C13 Z-enoate or the C9 OH (moieties responsible for the shallow orientation of PKC in the membrane, as in **10** or **11**), bryostatin-like activity is retained. When both are missing (as in **3**, simulated in this study), phorbol ester-like activity is instead retained. These are independent of PKCα-binding affinity

These effects agree with the results we observe with this model and membrane orientation. The coordination of waters to both the C13 Z-enoate and the C9 OH allows for a significant stabilization of the shallow PKC C1b–ligand–membrane complex. Removal of one of these functional groups cannot fully abrogate this stabilization; removing both, however, would eliminate stabilization of this shallow state, resulting in only one free energy minimum in the deeply embedded state, similar to the PDBu simulation. This is seen directly in the simulation of **3** (Fig. 3). These experimental data suggest that this stabilization of the shallow membrane orientation affects the activity of PKC as a whole and could be a reason for bryostatin-like activity. More generally and significantly, our studies indicate that more than one state of the PKC–ligand complex could contribute to activity. This represents a significant departure from conventional one model-one function analyses that are often based only on single state X-ray structures. Our model predicts that bryostatin analogs with water-coordinating moieties in the northern region will replicate bryostatin-like membrane association and potential activity, a prediction that allows a transition from screening to our ongoing hypothesis-based design of new ligands.

## Discussion

Elucidation of the structure and dynamics of membrane-associated proteins is an unsolved problem in both structural biology and chemistry. Less than 1% of crystal or solution NMR structures of proteins are of membrane-associated proteins^42,43^, and from this scarcity even less is known about peripheral membrane protein dynamics in a membrane environment. This limits an understanding of their activation and function and thus limits the design of new and more selective ligands to the use of information derivable only from single state, static crystal structures. While design based on a static crystal structure can often be effective, it also could be misleading or incomplete as crystal packing forces differ from membrane association in heterogeneity and dynamics. PKC is one such membrane-associated protein of intense current clinical interest, being evaluated clinically for the treatment of cancer and Alzheimer’s disease and the eradication of HIV/AIDS. Bryostatin, a potent PKC modulator, is currently in clinical trials for all three indications. Nevertheless, structural and dynamic information on a bryostatin–PKC C1b complex in a membrane environment is not known, thus hampering efforts to design more effective and accessible analogs. This information takes on added importance as the supply of bryostatin is uncertain due to its natural scarcity (14 tons of marine organism produced only 18 g of bryostatin)^44^. The total synthesis of natural bryostatins has progressed impressively but has not yet impacted clinical supply^29^. Thus, the design of simplified, more synthetically accessible, and more efficacious analogs has become a high priority and urgent goal.

We have reported bryologs that exhibit the same PKC affinity and selectivity as bryostatin but require much fewer steps to make^29^. However, no information, theoretical or experimental, exists on the structure and dynamics of bryostatin or any of these analogs in a membrane environment. The design of new ligands, especially those with high potency and high selectivity, depends critically upon this information. Significantly, for many ligands, affinity and activity might be a function of composite contributions from an ensemble of bound states and thus not revealed by a single X-ray structure^45^. We have indeed found that both the orientation of the peptide-ligand system in this membrane context and the shape of the overall thermodynamic landscape depend on the ligand. In other words, the ligand influences the positioning of the host–ligand complex in its microenvironment and in this case indicates the existence of two membrane-associated states. Proposed is a model attributing bryostatin’s ability in particular to change the PKC C1b-ligand orientation to the coordination of waters by its C9, C3, and C19 hydroxyls and its C13 Z-enoate. Such a model is plausible due to the unique hydration of the lipid headgroup region. In this region, solvation levels vary, wherein the amines at the end of the headgroups are fully solvated, and the fatty acid carbonyl groups are only slightly so. Only at the hydrocarbon region of the lipid is water completely absent. In its shallow PKC C1b-ligand-membrane state, bryostatin resides in this partially hydrated region. The aforementioned hydroxyls stabilize waters in the sparsely hydrated region near the lipid carbonyls, and the Z-enoate is stabilized by bulk waters outside the membrane. In both these cases, further penetration would incur significant desolvation penalties, creating the observed energetic barrier between the shallow and deep states.

Thus, the model described here presents bryostatin as holding the C1b domain in a different orientation in the membrane than other non-bryostatin-like ligands, lacking the necessary water-coordinating functionalities. Stabilizing one such orientation could influence its availability for interactions with other proteins whose phosphorylation is effected by PKC. There is a vast array of proteins that bind the C1 or nearby C2 domains, often selectively between isoforms^46–48^. Some affect the localization of PKC in different intracellular membranes, which is thought to be significant in distinguishing the functions of different PKC isoforms^49^. A different depth or angle of penetration of the C1a or C1b domains into the membrane can thus modulate PKC activation by dictating the availability of these protein–protein binding sites to their partners. It is plausible, then, that ligands that modulate this angle and depth in different ways could induce different PKC activity profiles.

The present work provides guidelines for the design of such ligands with an eye toward how they orient the PKC C1b–ligand–membrane complex. Most efforts thus far have focused upon retaining potency while simplifying the highly complex structures of the natural bryostatins, treating the PKC–ligand complex as a single contributor to activity. The present work provides testable hypotheses for what specifically makes bryostatin elicit unique activity that is distinct from other similarly potent ligands such as the phorbol esters, and acts as a guideline for future design and synthesis of simplified bryostatin analogs. It indicates that ligand activities could arise from the composite contributions of multiple ligand-host states, representing a transition from static X-ray-based analyses of single complexes to dynamic multi-state systems. Efforts are currently underway to experimentally explore these predictions using solid state REDOR NMR^24^ and to design and synthesize simplified bryostatin analogs that retain its water-coordinating moieties in order to test this hypothesis and replicate bryostatin-like function.

On a more technical level, this work demonstrates the power of MSMs for describing the structures and dynamics of complex systems. In previous studies, MSMs have been used to model the folding of proteins to a known native state^50^, or to demonstrate activation pathways and metastable intermediates between known active and inactive structures^51–54^. This study expands upon this body of knowledge by presenting a system in which the structure of the active state is experimentally inaccessible, and these MSMs resolve the active state through simulation alone. This is especially important in the realm of membrane proteins, given their therapeutic importance and the difficulty of obtaining experimental biorelevant structures, and doubly so for peripheral membrane proteins, whose membrane-associated states might be short-lived but individually or collectively critical to their function.

## Methods

### Structure preparation

The X-ray structure of the PKCδ C1b domain (PDB ID: 1PTR)^19^ was used as the starting point for all studies. Binding affinities of ligands to lone C1b domains have been shown to be comparable to those with the full-length protein^33^, and because there are unstructured regions separating the C1b domain from other structured domains on both its C and N termini, it is reasonable to expect that the absence of the other PKC domains will not dramatically affect the behavior of the peptide. Certain residues of the PKCδ C1b crystal structure were changed to model human PKC. The cysteines coordinating the C1b domain’s zinc ions were all deprotonated, and all other residues were protonated according to each one’s pK_a_ value at pH 7.4.

To obtain its bound pose, PDBu was overlaid with the structurally similar cocrystallized phorbol 13-acetate using ROCS^55,56^. The bryostatin 1 bound pose was found by first performing a conformational search using OMEGA^57,58^, docking these structures using FRED^59–61^, and using the highest scoring structure for further simulation (system preparation for prostratin and bryolog **3** is described below). Lipid parameters were derived from the Stockholm lipid (Slipid) parameters^62^. Comprehensive details on system setup and simulation parameters are provided in the Supplementary Methods.

### Simulation details

All simulations were performed using GROMACS 4.5.3 (ref. ^63^). The system was constructed by placing the PKCδ C1b domain adjacent to the membrane (with PDBu or bryostatin bound, or without ligand) such that the binding site is nearest to the lipid and the N and C termini are furthest from it. This system was pulled from solution into the membrane over ~200 ns of simulation time. Approximately 100 snapshots from each system were taken from along the pulled coordinate, equilibrated, and used as starting configurations for simulations on Folding@home. See Supplementary Figs. 8 and 9, and the Supplementary Discussion for a discussion of the impact of these starting configurations on the final results. In total, the PDBu, bryostatin 1, and ligand-free systems each had approximately 400 µs aggregate simulation time and average trajectory length of 30 ns. For the prostratin simulations, the PDBu starting structures were directly modified, converting the C12 butyrate to a hydrogen and the C13 butyrate to an acetate. For the bryolog **3** simulations, the bryostatin starting structures were directly modified, converting the C8 *gem*-dimethyl and C9 OH to hydrogens, and the C13 Z-enoate to an exocyclic pyran olefin. In for both prostratin and bryolog **3**, these changes had no impact upon ligand conformation. The resulting structures were minimized and simulated directly on Folding@home. The prostratin simulations had aggregate simulation time of 580 µs and average trajectory length of 115 ns. Bryolog **3** had aggregate simulation time of 200 µs and average trajectory length of 15 ns.

### Data analysis

Each data set was featurized using MSMBuilder 3 (ref. ^64^). Featurization is the process by which certain quantities particular to a system (such as the dihedral angles of the protein backbone) are aggregated from every relevant atom and every frame. This data (the system’s “features”) act as a way to describe the structure and dynamics of the system as a whole. Seven different featurizations were used to describe the system in this study. Three of these measured protein conformation in different ways (backbone dihedral angles, r.m.s.d. of protein when superposed with a reference structure, and distribution of reciprocal interatomic distances of all heavy atoms). Two measured the localization of water around the protein (the solvent shell featurizer^65^ measures the local, instantaneous water density around all protein and ligand heavy atoms within a certain radius; in this case radii of 0.3 and 1.0 nm were used). Two measured the localization of lipid molecules around the protein and ligand (one used the same solvent shell featurizer except using lipids instead of waters, and the other used a weighted metric measuring the distance of every protein heavy atom to every lipid phosphorus atom)^66^. These seven featurizations were combined such that each new one consisted of 0 or 1 characterization of the protein, water, and lipid systems. This yielded a total of 35 different featurizations for future analysis.

On each of these featurizations, we performed a tICA analysis^67^. We measured the four most slowly decorrelating linear combinations of these features, using a tICA lag time, *t*, between 0.5 and 5 ns and an L2 regularization strength gamma, *g*, between 10^−1^ and 10^−10^. These tICs were then clustered into *k* states using the mini-batch *k*-means clustering method^68^. Using this clustering, MSMs were built using lagtime of 4.5 ns (see Supplementary Fig. 10 for the implied timescales plots of these MSMs). In order to determine optimal values for *t*, *g*, and *k* of the model, we sought to maximize the eigenvalues of our MSM, in accordance with the variational formulation of kinetics introduced by Nüske and Noé^69^. However, we were also cognizant that overfitting, arising, for example, due to an overly large value for *k* or overly small of a value for *g*, could pose a risk. For this reason, we selected these values by cross-validation using the variational GMRQ objective function described by McGibbon and Pande^70^, which assesses how well the MSMs maximize a variational criterion evaluated on data that was held out during the fitting of the model. This optimization was managed by osprey (available at https://github.com/pandegroup/osprey), a tool for hyperparameter optimization of algorithms in machine learning.

Each iteration of the optimization involved building an MSM using a random subset of the data (the training set) and evaluating how slowly the first six eigenvectors of the MSM decorrelate when measured against the remaining subset of the data (the training set). Those that decorrelate the most slowly have the highest GMRQ scores and can be considered the “best” MSMs. Projections shown in Fig. 3 show histograms representative of the top five GMRQ-scoring featurizations of each of the PDBu, bryostatin, bryolog **3**, prostratin, and ligand-free systems (see Supplementary Fig. 11 for a full listing of the top five GMRQ-scoring projections for each system).

### Data availability

The simulation data that support the findings of this study are available from the corresponding author upon reasonable request.

## Electronic Supplementary Material

Supplementary Information Supplementary Figures, Supplementary Discussion and Supplementary References.
