# Supplementary material for: Molecular dynamics simulations reveal ligand-controlled positioning of a peripheral protein complex in membranes
Source: Nat Commun. 2017 Feb 23;8:6. doi: 10.1038/s41467-016-0015-8 (PMC5431895; doi:10.1038/s41467-016-0015-8)
Supplement: Supplementary file 1 — Supplementary Figures, Supplementary Discussion and Supplementary References. [file 41467_2016_15_MOESM1_ESM.pdf]

Supplementary Figures

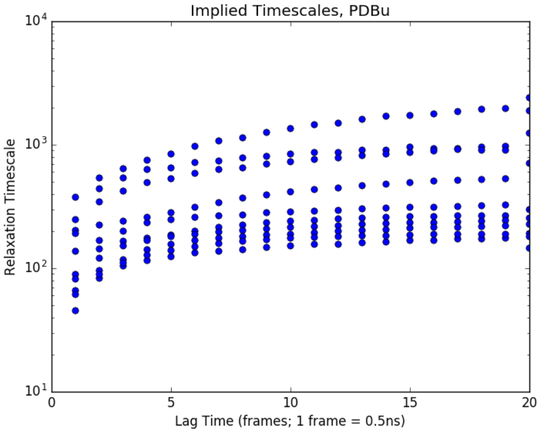

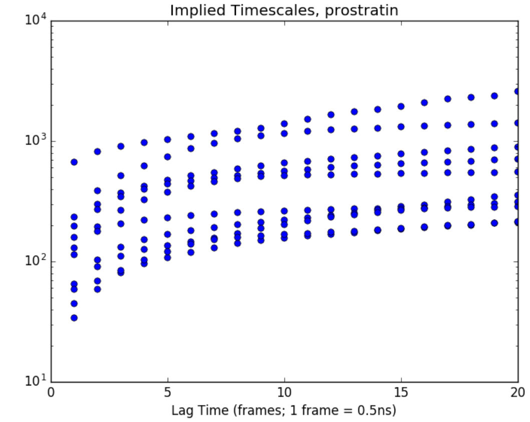

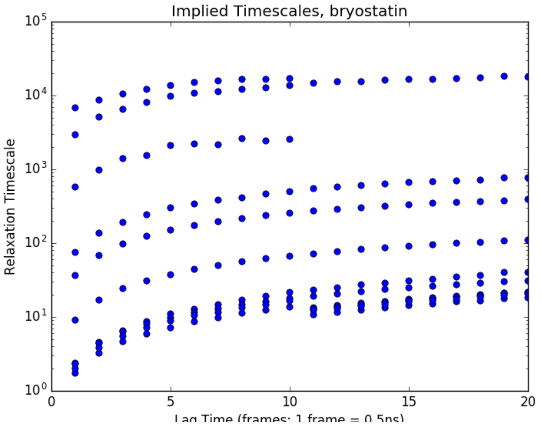

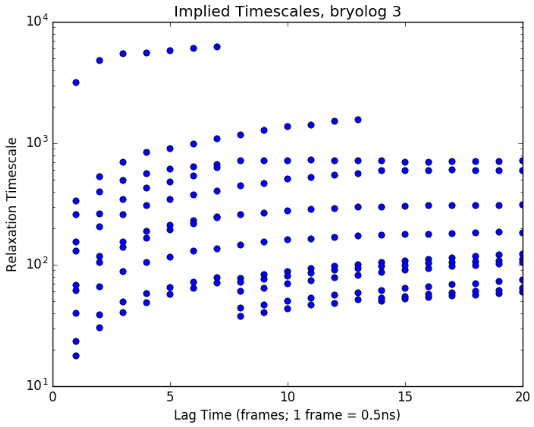

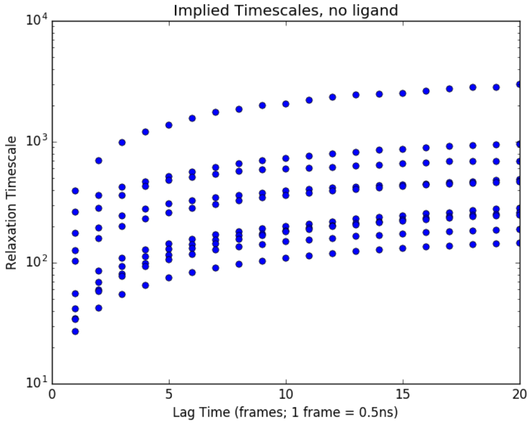

Supplementary Figure 1: Implied timescales plots for all systems. x-axis corresponds to lag time frames, where each frame is 0.5 ns. See Supplemental Methods section on Data Analysis: Markov State Models for discussion of these implied timescales.

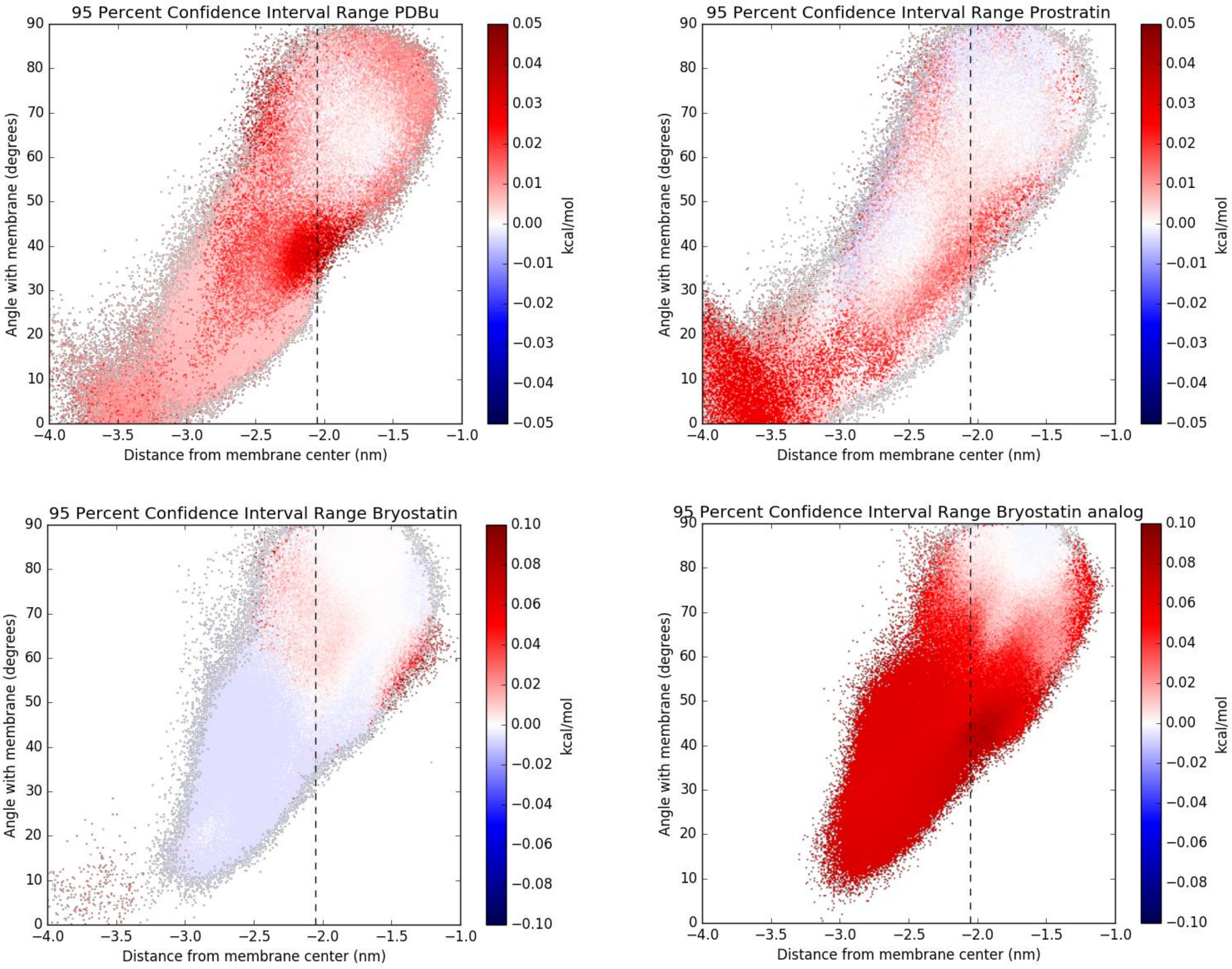

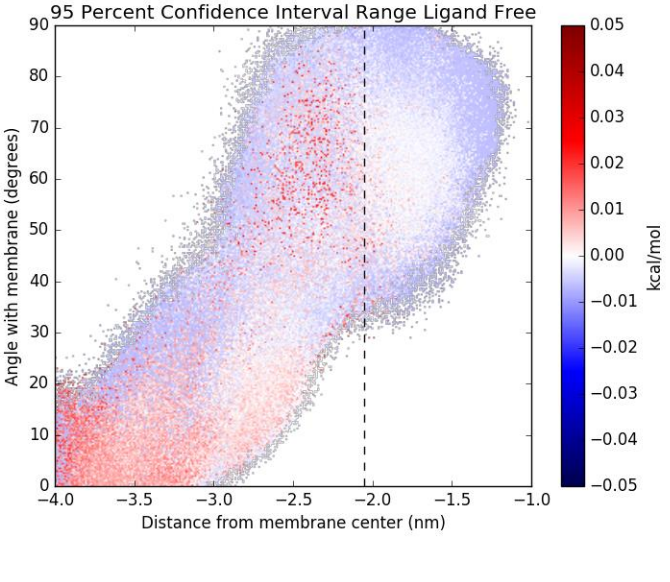

Supplementary Figure 2: Range of 95% confidence intervals of the free energy for PDBu, prostratin, bryostatin, bryostatin analog **3**, and no ligand, where distance 0 is the plane through the center of the membrane. Note that even for the most extreme example (bryostatin analog **3**, lower right), the greatest extent of the 95% confidence interval is merely 0.10 kcal/mol. See Error Analysis and Bootstrapping section of Supplemental Discussion for more information.

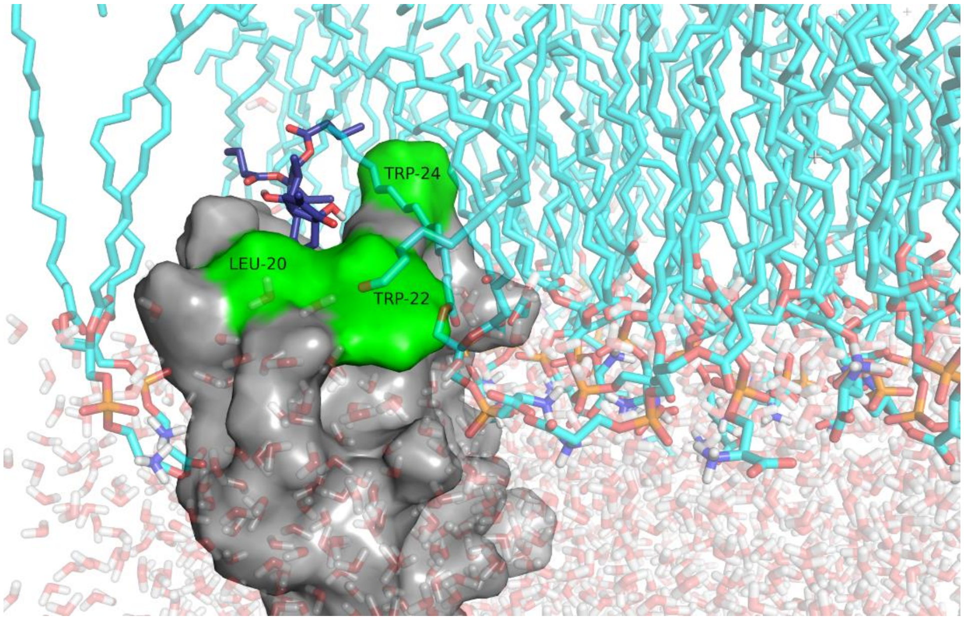

Supplementary Figure 3: Snapshot of PKCδ C1b-PDBu system associated with the membrane in its lowest free energy configuration. The three hydrophobic residues posited to be embedded in the hydrophobic region of the membrane are labeled in green. Note that these residues are indeed localized in the hydrophobic region of the membrane.

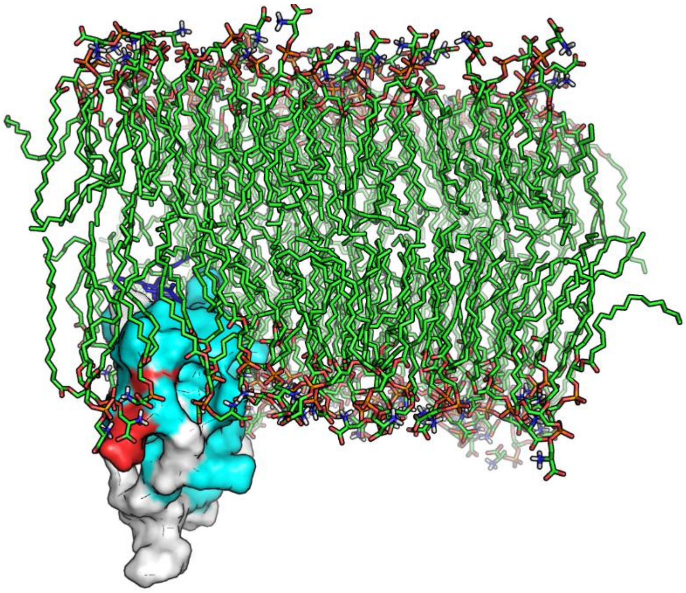

Supplementary Figure 4: Representative snapshot of a state in the free energy minimum of the PKCδ C1b-PDBu complex with the membrane. Cyan surface indicates residues whose solution NMR signals broadened in the presence of lipid. Red surface indicates residues whose signal broadened only in the presence of lipid AND PDBu. Note that all cyan residues are in contact with or near to the membrane. Note also that the red residues (the ones that only broaden in the presence of PDBu) are shallowly embedded near the headgroups, suggesting that PDBu causes these residues to more closely associate with the membrane than in the absence of ligand.

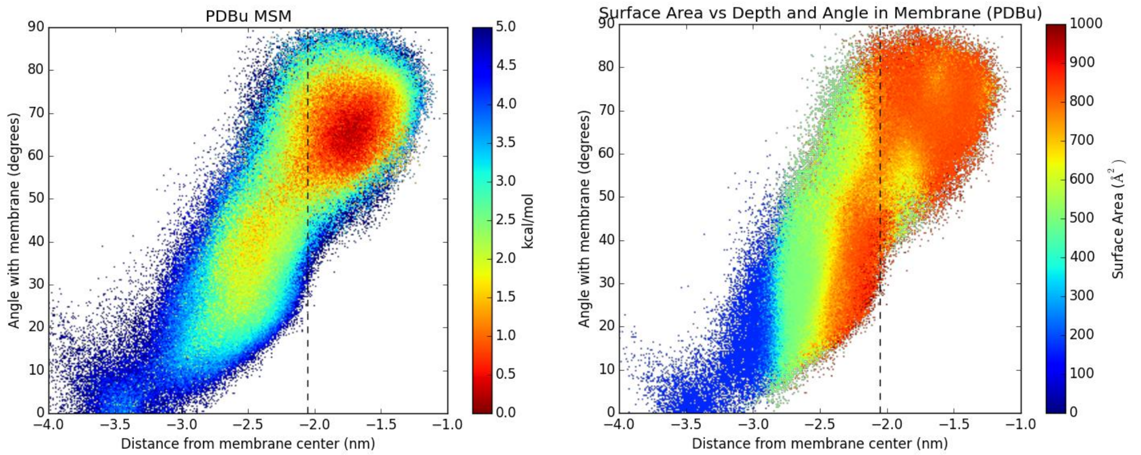

Supplementary Figure 5: Left: Free energy histogram of PDBu vs angle and depth of peptide penetration. Right: surface area in headgroup region of membrane vs angle and depth of peptide penetration. The headgroup region is defined as the region between the average position of the second carbon on each lipid acyl chain and the average position of the nitrogen atom on the headgroup. Note that the free energy minimum of the PDBu MSM coincides with a region on the surface area plot well within the calculated 840 ± 42 Å^2 range.

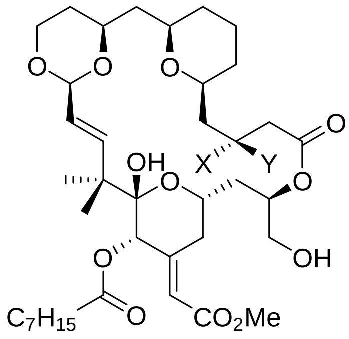

S1: X = OH, Y = H, PKC K_i = 3.4 nM
S2: X = H, Y = OH, PKC K_i = 285 nM
S3: X = H, Y = H, PKC K_i = 297 nM

Supplementary Figure 6: Activity series of C3-modified bryostatin analogs. epi-C3 and deoxy-C3 analogs **S2** and **S3** show greatly diminished activity compared with analog **S1**.

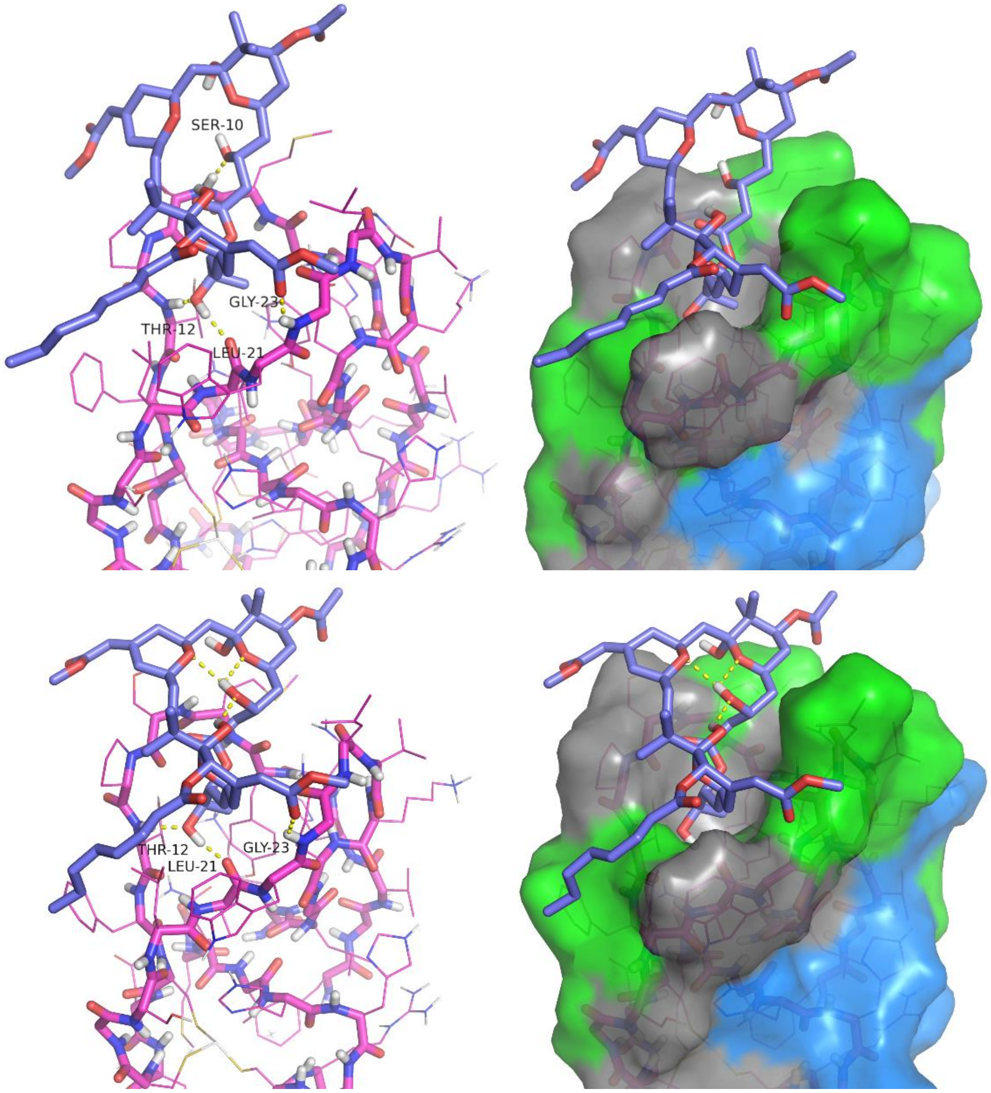

Supplementary Figure 7: Bound poses of bryostatin. Top: Most prominent bryostatin conformation in simulation. This is notable for its hydrogen bond between the C3 OH and SER-10, as well as several hydrogen bonds between these moieties and waters (see Figure 4 in the main text). Bottom: Commonly suggested bound pose of bryostatin, possessing an internal hydrogen bond between the C19 OH, the C3 OH, and the A- and B-rings. In both surfaces shown, green indicates hydrophobic residues, blue indicates basic residues, and gray indicate those that are neither.

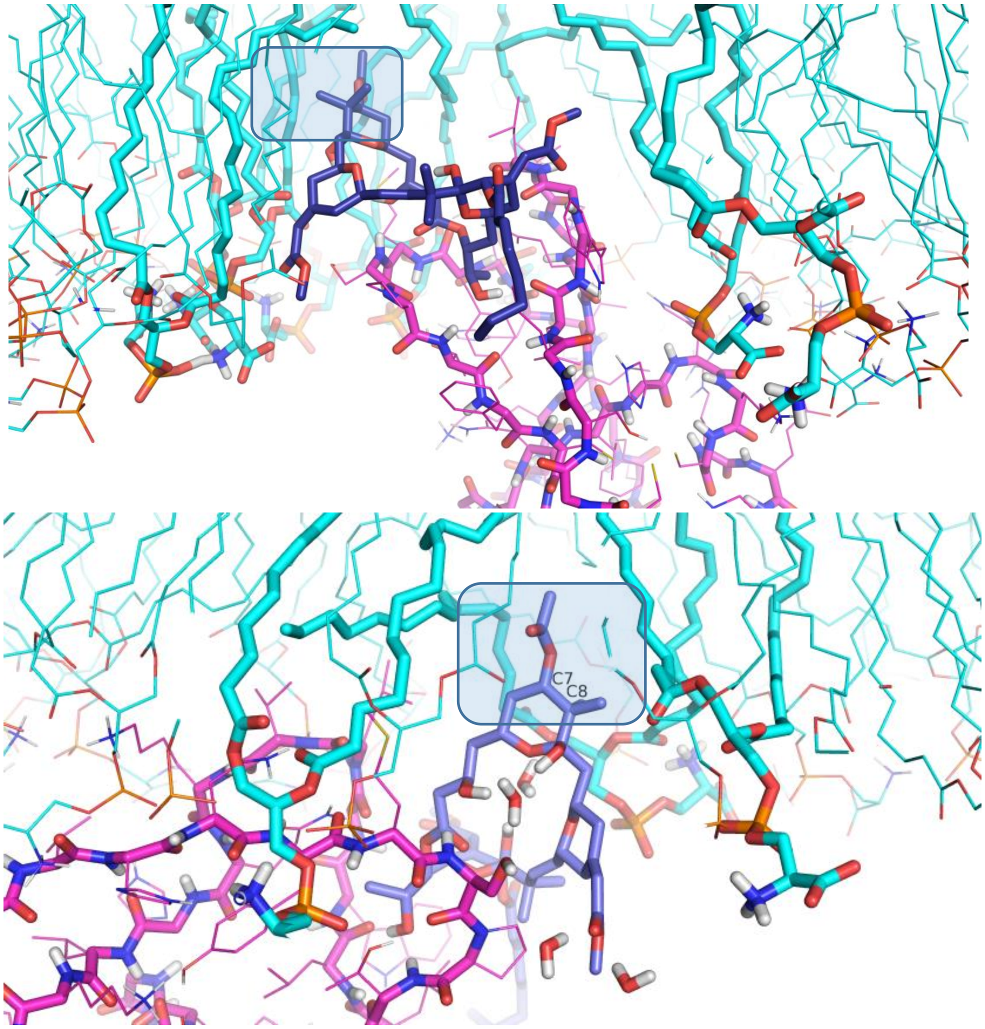

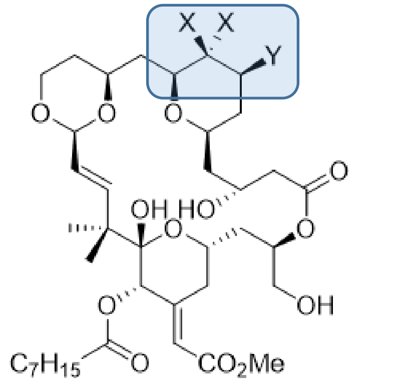

S4: X = H, Y = H, PKC K_i = 0.25 nM
S5: X = Me, Y = OH, PKC K_i = 19nM
S6: X = H, Y = OH, PKC K_i = 1000nM

Supplementary Figure 8: Top left: PKCδ C1b-bryostatin-membrane complex in deep orientation (waters not shown). Bottom left: PKCδ C1b-bryostatin-membrane complex in deep orientation (only waters coordinated by bryostatin shown). Note the C8 *gem*-dimethyl and C7 acetate as the most deeply membrane-embedded functionalities in both cases (highlighted). In all snapshots examined, the C7 acetate is never coordinated by waters, and in most cases, even in the shallow configuration, these two groups are within the hydrocarbon region of the membrane. Right: Bryostatin analog binding affinity depends on lipophilicity of this northern region. When both C7 and C8 are unsubstituted, binding affinity is very strong (**S4**). When C7 is hydroxylated but C8 is unsubstituted, affinity dramatically weakens (**S6**). When C8 has a *gem*-dimethyl and C7 is hydroxylated, much binding affinity is recovered (**S5**). This agrees with the model, where these functionalities are deeply embedded in the hydrophobic core of the membrane.

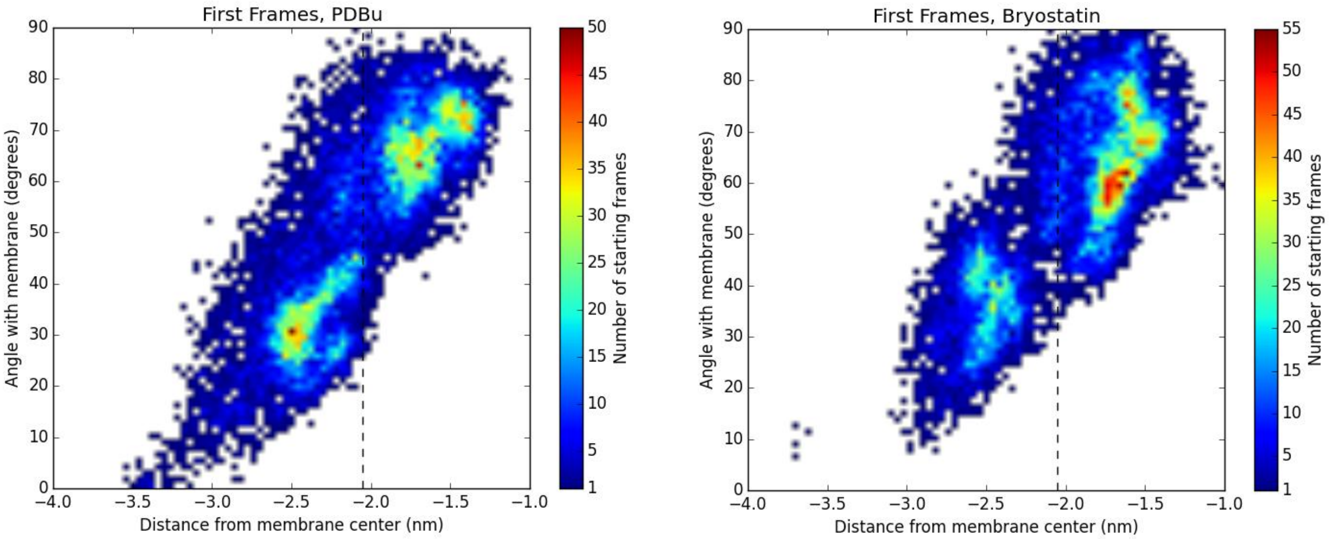

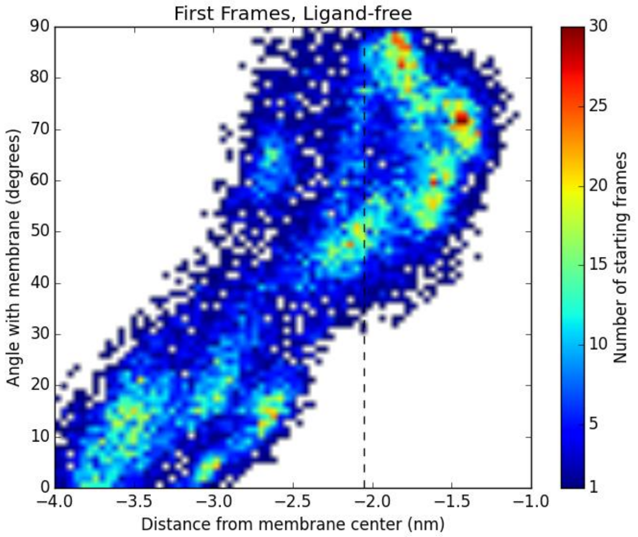

Supplementary Figure 9: Histograms showing distribution of the starting frames of the PKCδ C1b-ligand system in the membrane. While there is a relatively flat distribution given the number of different trajectories (~10,000 per system), it is still not entirely uniform, and there still must be some test for whether these starting configurations bias the system (see **Supplementary Figure 10** below).

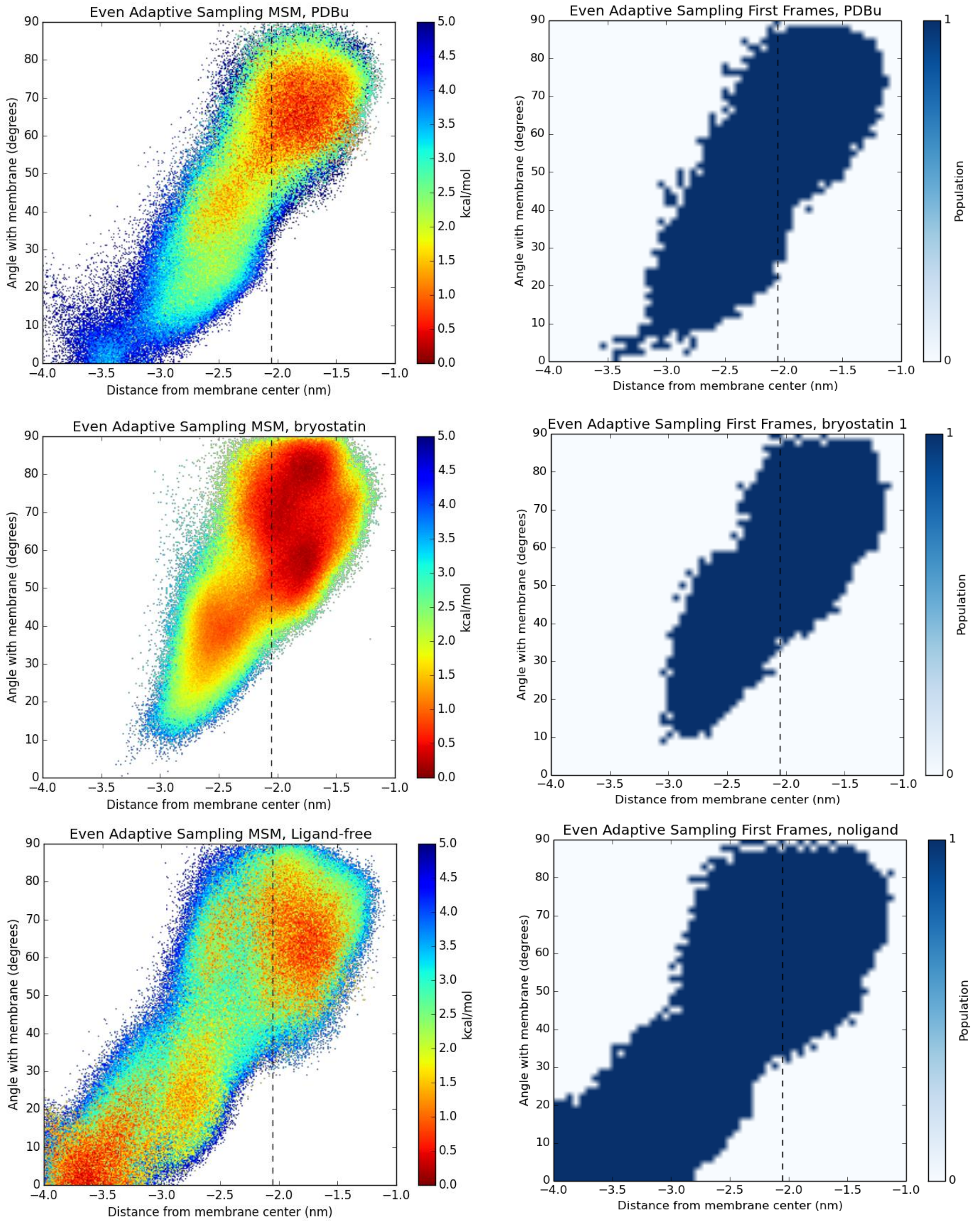

Supplementary Figure 10: Left: Free energy histograms of the PKCδ C1b domain with PDBu (top) or bryostatin (middle) bound, or without a ligand (bottom), from simulations with starting configurations that are entirely evenly distributed. Right: Plot of starting configurations as a function of angle and depth in the membrane, where each blue square indicates precisely one starting configuration with that orientation. Note that the free energy histograms on the left are virtually identical to those with made with the full dataset (see also Figure 3 in the main text for histograms of the full datasets).

Supplementary Figure 11: The free energy histograms for the five top GMRQ-scoring MSMs are shown below for each system.

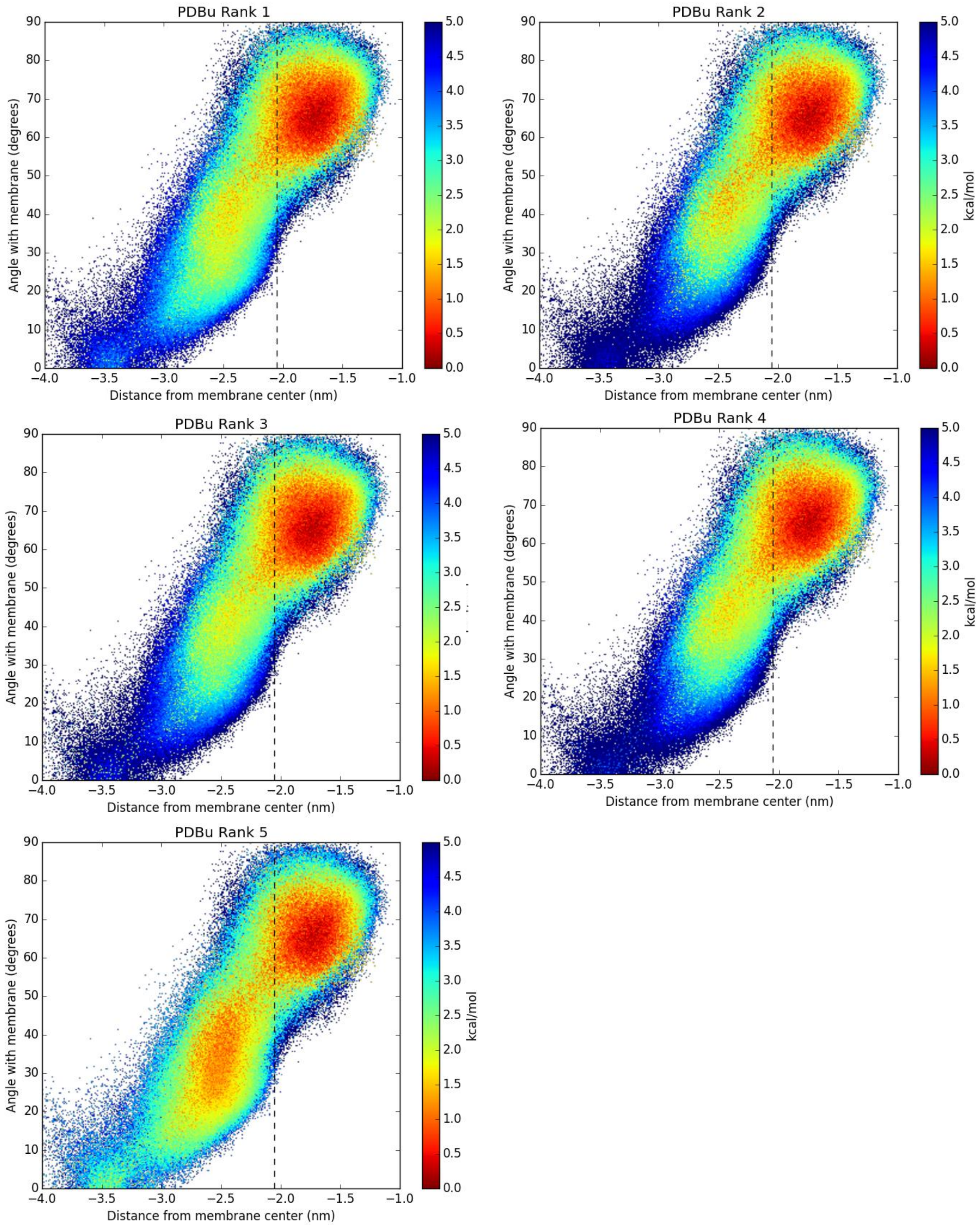

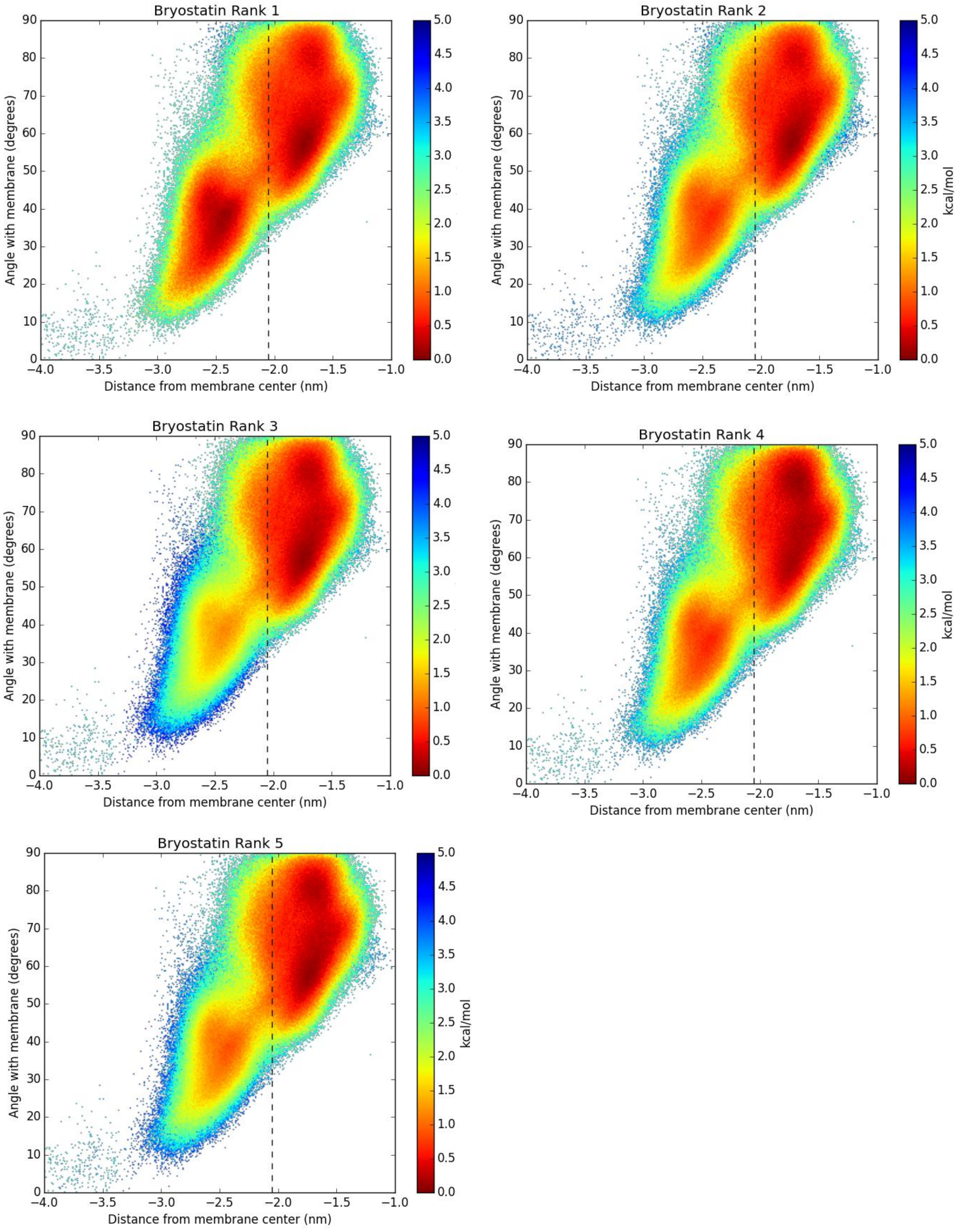
Bryostatin Rank 1
Bryostatin Rank 2
Bryostatin Rank 3
Bryostatin Rank 4
Bryostatin Rank 5
Angle with membrane (degrees)
Distance from membrane center (nm)
kcal/mol
0
10
20
30
40
50
60
70
80
90
−4.0
−3.5
−3.0
−2.5
−2.0
−1.5
−1.0
0.0
0.5
1.0
1.5
2.0
2.5
3.0
3.5
4.0
4.5
5.0

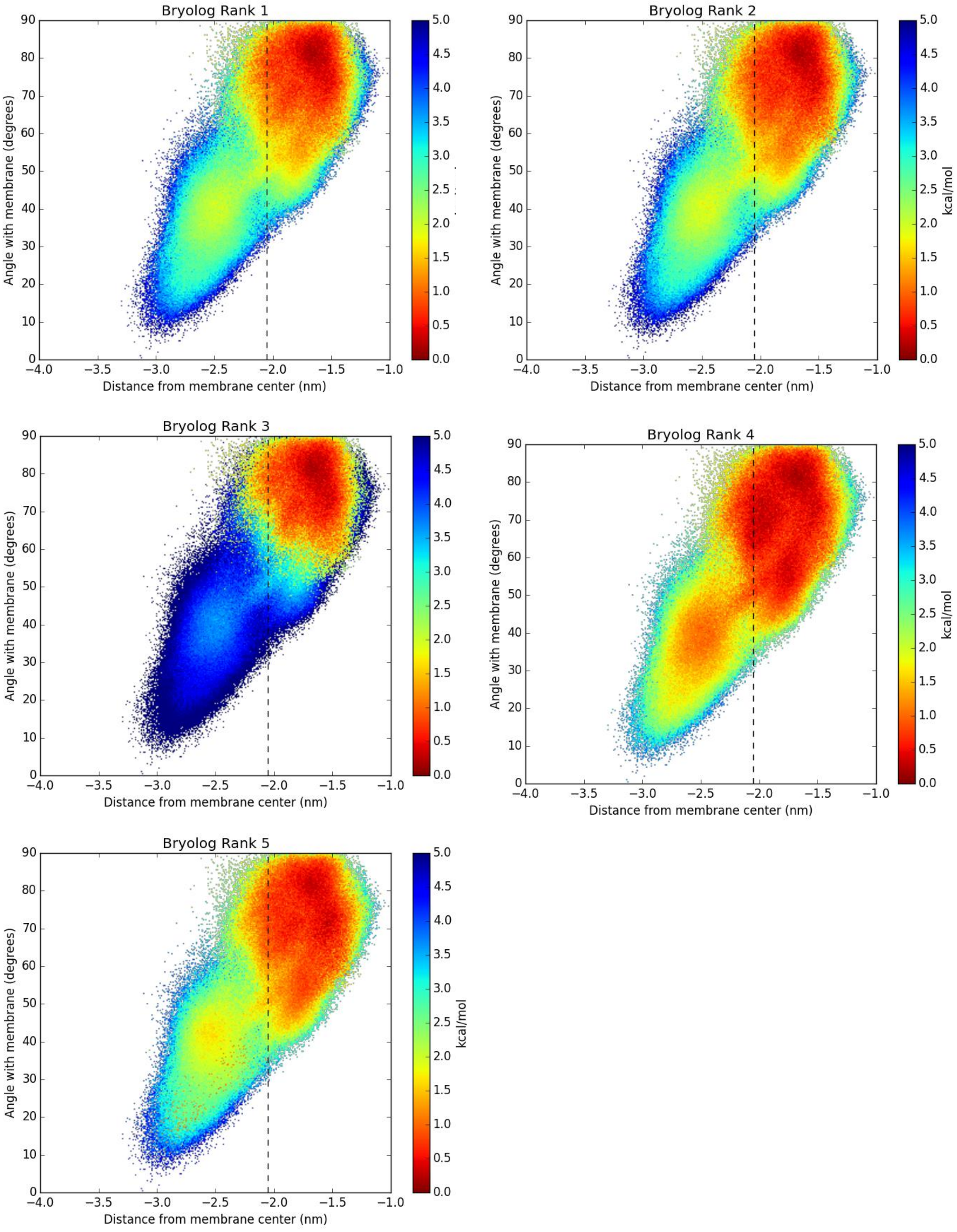
Bryolog Rank 1
Bryolog Rank 2
Bryolog Rank 3
Bryolog Rank 4
Bryolog Rank 5
Angle with membrane (degrees)
Distance from membrane center (nm)
kcal/mol

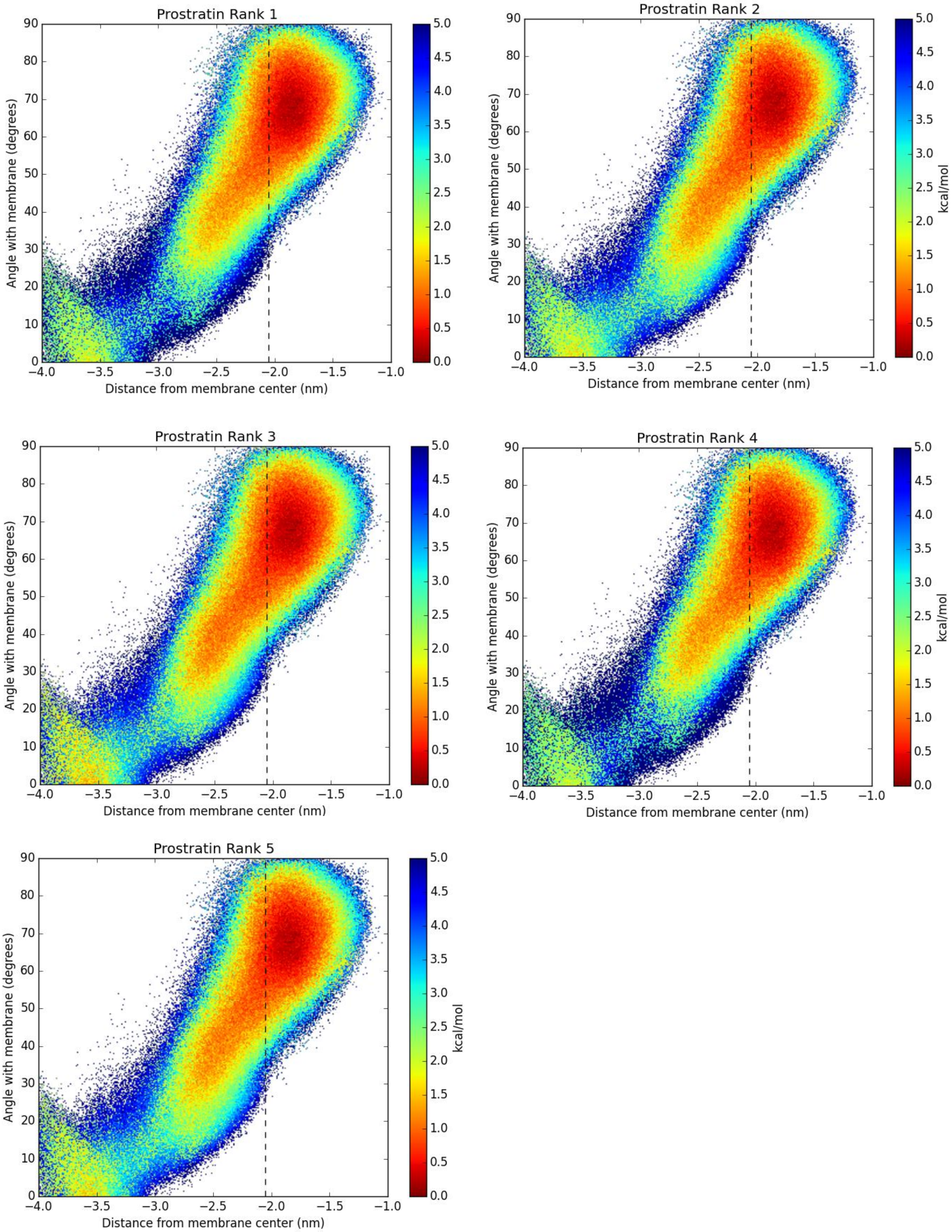
Prostratin Rank 1
Prostratin Rank 2
Prostratin Rank 3
Prostratin Rank 4
Prostratin Rank 5
Angle with membrane (degrees)
Distance from membrane center (nm)
kcal/mol
−4.0 −3.5 −3.0 −2.5 −2.0 −1.5 −1.0
0 10 20 30 40 50 60 70 80 90
0.0 0.5 1.0 1.5 2.0 2.5 3.0 3.5 4.0 4.5 5.0

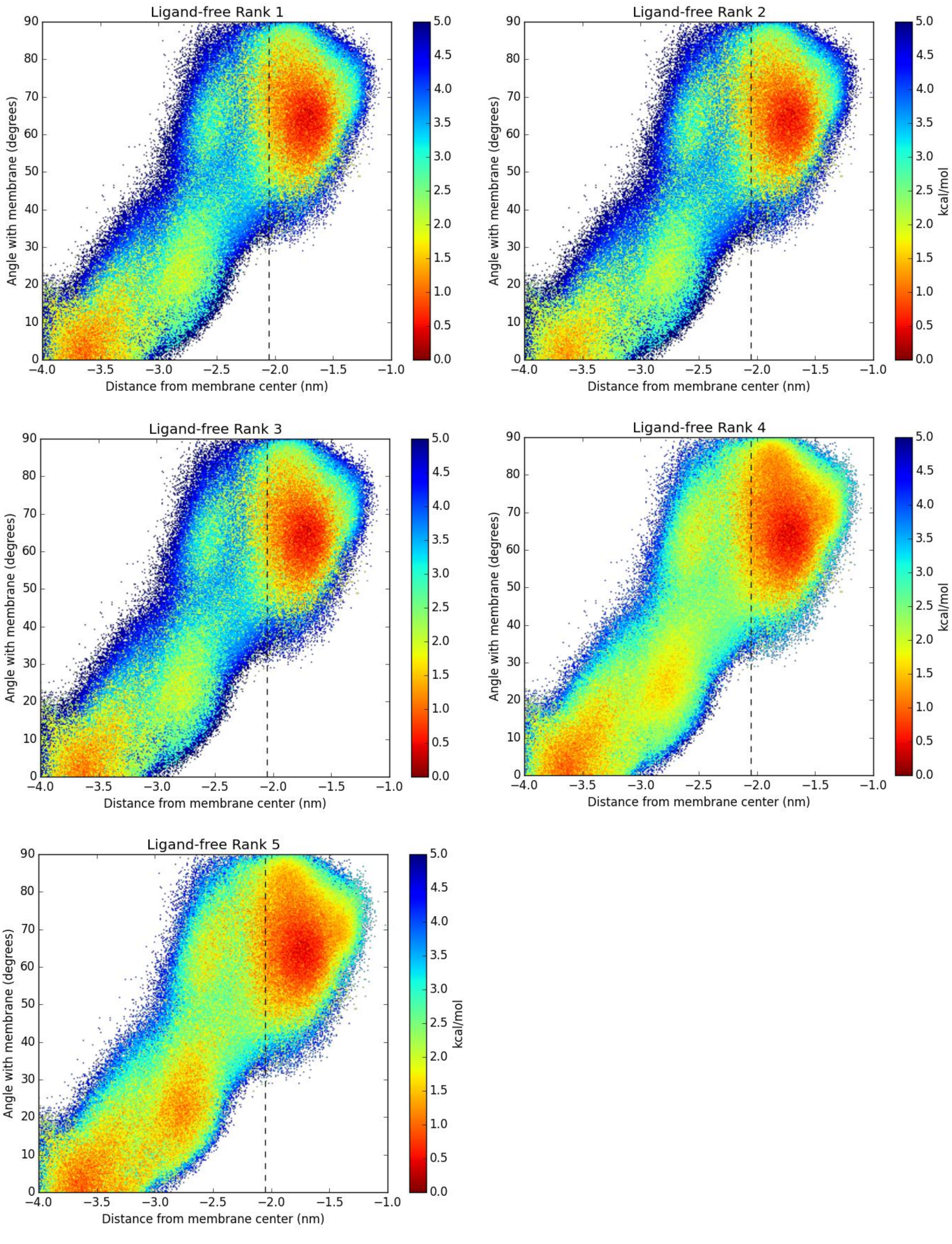
Ligand-free Rank 1
Ligand-free Rank 2
Ligand-free Rank 3
Ligand-free Rank 4
Ligand-free Rank 5
Angle with membrane (degrees)
Distance from membrane center (nm)
kcal/mol
0
10
20
30
40
50
60
70
80
90
−4.0
−3.5
−3.0
−2.5
−2.0
−1.5
−1.0
0.0
0.5
1.0
1.5
2.0
2.5
3.0
3.5
4.0
4.5
5.0

Supplementary Discussion

Error analysis and bootstrapping

An important effort to consider is the statistical significance of the energies on the free energy histograms in the main text. In an effort to probe this, bootstrapping simulations have been performed on each system in order to ascertain the 95% confidence interval for free energy across the landscape. This is done to ensure that the free energy of a given state is not merely due to a single rogue trajectory but is instead representative of the entire ensemble. One could imagine a system in which a single long trajectory becomes "stuck" in a given state such that it would dramatically lower the free energy of that state while not being truly representative of the ensemble. If such a scenario were to occur, there would be a very broad 95% confidence interval.

These bootstrapping simulations have revealed that the 95% confidence interval of each system is extremely narrow across all sampled points in space. The maximum difference from the lower bound and the upper bound of this confidence interval is 0.05 kcal/mol for the PDBu, ligand-free, and prostratin systems, and 0.1 kcal/mol for the bryostatin and bryolog 3 systems. This indicates that the reported $\Delta\Delta G$ of 1 kcal/mol between the "shallow" and "deep" configurations between bryostatin and bryolog 3 discussed in the main text is highly significant. The histograms plotting the range of the 95% confidence intervals of the free energy of all five simulated systems are shown in Supplementary Figure 2.

Lowest free energy membrane configuration agrees with experimental evidence

There have been several efforts to probe the structure of the membrane-associated state of the ligand-bound PKC C1b domains. In all cases thus far, the experimental evidence for this membrane-associated structure agrees with the observed free energy landscape of the PKC-ligand-membrane systems in our simulations. Several of these efforts, and their comparison with our simulations, are described below.

Mutation Study

One effort to probe the structure of the membrane-associated PKCδ C1b domain examined the effect of mutation of its putatively membrane-embedded residues. The ligand binding site of the PKCδ C1b domain is surrounded by several hydrophobic residues (M9, F13, L20, L21, W22 G23, L24, and V25). These are presumed to thermodynamically favor the insertion of the protein-ligand complex into the hydrophobic region of the membrane.

Wang *et al.* found in this study that, near the binding pocket, the mutation of any of three neutral residues to basic ones (L20R, W22K, L24K) abrogated ligand binding in the presence of lipid, but had a much smaller effect upon binding in the absence of lipid.[1] The cationic residues did not significantly affect the ligand-protein interactions, but their presence in the hydrophobic membrane is be highly unfavorable. The conclusion is that these residues are likely localized deep in the phospholipid membrane, away from the highly charged headgroup region.

These results agree with the PDBu model developed herein, where these three residues are all embedded in the phospholipid membrane in the deeply-embedded lowest free energy state. An example snapshot of the PKCδ-PDBu system in this low free energy state is shown in Supplementary Figure 3.

Solution NMR study

A solution NMR study was performed by Xu, *et al.* that examined which residues of the PKCγ C1b-PDBu system are embedded in the membrane.[2] It was observed that peaks for 29 of the 50 residues of the PKCγ C1b domain broaden upon addition of phosphatidylserine vesicles, and an additional 3 broaden when PDBu is added as well. This indicates that these residues are closely associated with the membrane. It is reasonable to assume that PKCδ, the isoform simulated in this study, is embedded in the membrane similarly to PKCγ due to the high homology of their C1b domains (64% sequence identity and 80% sequence similarity). Of the 32 residues that broaden upon addition of vesicles and PDBu in the PKCγ system, every corresponding PKCδ residue is closely associated with the membrane in our simulations. In the system's lowest free energy state, these residues are either deeply embedded within the membrane or localized at the water-membrane interface. This is shown in Supplementary Figure 4.

Calculated Surface Area

In a study performed by Ziemba and Falke,[3] the above NMR experiment was used to calculate the surface area of the C1b domain embedded in the headgroup region of the membrane (defined by the space between the average positions of the second carbon on the lipid acyl chains and the nitrogens on the lipid headgroups). This value was calculated to be 840 ± 42 $Å^2$. This value agrees very well with these simulations. The surface area of protein embedded in the headgroup region of the membrane was calculated for every frame of simulation using the method developed by Shrake and Rupley[4], and is plotted as a function of angle and depth in the membrane in Supplementary Figure 5. Notably, the region of lowest free energy for the PDBu-PKCδ C1b system coincides with a region on the surface area plot well within the calculated 840 $\pm$ 42 $Å^2$ range.

Bryostatin 1 has several bound poses

In 1986, we first described a model claiming that all PKC modulators contain a triad of hydrogen bond donors and acceptors.[5] Both x-ray crystallography[6] and solution NMR[7] experiments have suggested that bryostatin possesses an intramolecular hydrogen bonding network, wherein the C19 hemiketal OH is hydrogen bonding with the C3 alcohol, which, in turn, acts as a hydrogen bond donor towards the A- and B-ring pyran oxygens. This results in a preorganized ligand with a pharmacophoric triad that is correctly spatially arranged for binding to the C1b pocket. It has been observed that *epi*-C3 and deoxy-C3 analogs (**S2** and **S3**, respectively; see Supplementary Figure 6) demonstrate severely abrogated activity, with the explanation that modifying this functional group disrupts the internal hydrogen bond network, thus making the bryostatin bound pose more energetically unfavorable.[8]

The simulations performed on bryostatin in this study add depth to this structural hypothesis. While we do observe this preorganized conformation in our simulations, we also observe describe several other conformations in which bryostatin's internal hydrogen bond is disrupted. The most prominent of these is shown in Supplementary Figure 7 with waters and lipids removed for clarity. Indeed, the most dominant conformation appears to feature the C3 OH hydrogen bonding with the Ser-10 amide proton, as well as with a structured water shared with the C9 OH and the Ser-10 side chain OH (see Figure 4 in the main text). It is plausible that the abrogated activity of the *epi*-C3 and deoxy-C3 compounds *is* due to a loss of preorganization, but that upon binding with this preorganized structure, bryostatin undergoes a conformational change to the more favorable pose observed in this model. It is also possible that the abrogation of activity is not due to preorganization at all, but instead simply due to loss of the highly favorable hydrogen bond network involving S10, C3 OH, C9 OH, and the water shared between all of them.

Separately, it is important to note that if C3 is engaged in an internal hydrogen bond with the C19 OH and the A- and B-rings, it cannot hydrogen bond with a water as shown in Figure 4 in the main text. Even in these situations, however, the C9 OH remains hydrogen bonded with a water when in the shallowly associated membrane orientation. In either case, the C13 Z-enoate remains able to hydrogen bond with waters as well. Thus, regardless of bryostatin's conformation, waters offer significant stabilization of the PKC-bryostatin complex when shallowly associated with the membrane.

Depth of PKC-ligand complex into hydrophobic region of membrane modulates ligand binding affinity

There are several moieties on bryostatin where small changes to the structure induce large changes to binding affinity. One such example concerns the effect of C7 and C8. When both these atoms lack substitution, as in **S4** (see Supplementary Figure 8), the bryolog binds strongly to PKC. When C7 features an OH (**S6**), this binding affinity is dramatically abrogated. When C7 features an OH *and* C8 features a *gem*-dimethyl (**S5**), however, much of this affinity is recovered. This has been previously explained with solvation: insertion into the membrane is heavily disfavored for a secondary alcohol due to a high desolvation barrier, but when adjacent to a *gem*-dimethyl group, the prior solvation of the alcohol is lessened, and the hydrophobic effect contributing to insertion is enhanced.[9,10]

The model described here provides a structural analysis of this suggestion. In virtually all configurations, the angle of the PKC-bryostatin-membrane system is such that the C7 acetate is the most deeply embedded moiety of the entire system. Even in the shallow, water-stabilized state, both the C7 acetate and C8 *gem*-dimethyl are localized in the hydrocarbon region on the membrane, and the C7 acetate lacks any water coordination (Supplementary Figure 8). It is thus reasonable to expect that if this acetate were instead an alcohol, there would be a large desolvation penalty to pay in order to reach the same membrane associated state we observe as the free energy minimum of the system. Wang *et al.* have observed that ligand binding to the PKCδ C1b domain is significantly weaker in the absence of a phospholipid membrane.[1] Thus, by precluding insertion into the membrane, the presence of this C7 OH will significantly diminish binding affinity, and this effect will be lessened when adjacent to a lipophilic moiety.

These simulations add depth to this previously-observed effect by demonstrating the localization of the C7 moiety specifically in the hydrophobic region of the membrane.

Adaptive sampling simulations demonstrate a converged system

It is important to examine the starting configurations of all the simulations in order to ensure that the free energy landscapes shown are real and not simply the result of a system biased by its starting states. As shown in Supplementary Figure 9, the histograms of the starting configurations for all simulations is not completely flat, and it is important to demonstrate such a starting landscape is not unreasonably biasing the system towards a certain conclusion.

In order to ascertain whether these starting configurations were the reason for the results of the MSM histograms, adaptive sampling simulations were performed. In these simulations, approximately one thousand starting configurations were chosen for the bryostatin, PDBu, and ligand-free systems such that each one was evenly spaced by depth and angle with regards to the membrane (see Supplementary Figure 10). These simulations were performed with approximately 1000 starting frames and four clones per starting frame. From these new starting configurations, PDBu and the ligand-free simulations had an average trajectory length of approximately 40 ns for PDBu and ligand-free, while bryostatin had an average trajectory length of 78 ns. These simulations were built into MSMs using same methods as described in the methods section. This resulted in MSMs that have energetic landscapes very similar to the simulations of the entire system. Such a result indicates that the starting configurations do not have a dramatic impact upon the energetics and shape of the MSMs that have been built to describe the system's free energy landscape, and that the results described in this paper are robust and converged.

Notably, such an effort was unnecessary for the prostratin and bryolog **3** simulations, as the starting configurations for these systems, prepared as described above, were made directly from the evenly-distributed adaptive sampling starting configurations of PDBu and bryostatin, respectively. Thus, the starting configurations of all prostratin and bryolog **3** simulations were evenly distributed across the thermodynamic landscape, and the simulations described in this paper are unbiased with regards to starting configuration.

Top GMRQ-scoring MSMs for each system

The free energy histograms shown in Figure 2 in the main text are representative of the top GMRQ-scoring MSMs of each of these systems. A full listing of the top five GMRQ-scoring MSM free energy histograms is shown in Supplementary Figure 11. These were obtained by using different featurizations as described in the methods sections, then ranking the subsequent MSMs by GMRQ score. In virtually all cases, the top MSMs had similar free energy-landscapes, and so one of these, representative of the whole, is shown in Figure 2. The primary

exception is in the rank 3 GMRQ-scoring MSM of bryolog **3**, which shows an extraordinary difference in free energy between the deep and shallow states. This appears to be an outlier and as such, a histogram representative of the other top four MSMs is used in the main paper. It must be noted, though, that, although this histogram is extreme, it does embody the same trend that is discussed in the main paper, wherein bryolog **3** lacks bryostatin's water-coordinating moieties, and thus prefers to be deeply embedded in the membrane rather than associated in a shallow way.

Supplementary Methods

Structure Preparation

The x-ray structure of the PKCδ C1b domain (PDB ID: 1PTR)[11] was used as the starting point for all studies. The C1b domain is the primary region of interest for probing the impact of PKC activators, and binding affinities of ligands to lone C1b domains folded in buffered water in the presence of phosphatidylserine vesicles have been shown to be comparable to those with the full-length protein.[12] Furthermore, because there are unstructured regions separating the C1b domain from other structured domains of PKC on both its C and N termini, it is reasonable to expect that the absence of the other PKC domains will not dramatically affect the behavior of the C1b peptide.

In the PKCδ C1b crystal structure, side chains of Lys-4, Arg-43, and Glu-44 were omitted from the structure due to their mobility. These side chains were restored for these simulations. The crystal structure was also that of mouse PKC; in order to simulate the human PKC, a single residue change was performed (Y6H). Finally, in order to avoid artifacts resulting from the charges of the C and N termini, two additional residues from the sequence of full-length PKCδ were added to both ends (Met and Pro on the C terminus, with a negatively charged C terminus, Gly and Ile on the N terminus, with a positively charged N terminus). The N-terminal residue was protonated, and C-terminal residue was deprotonated. The structure used was a homology model incorporating these changes using MODELLER.[13] The six cysteines coordinating the two zinc ions were made to atom type CYM (as opposed to CYS), indicating a deprotonated side chain. Furthermore, harmonic springs were used to connect the six cysteines and two histidines to these zincs to force coordination and preclude zinc escape into solution. The zinc does not remain coordinated without these restraints, as this model lacks polarizability or quantum effects required to ensure proper metal coordination. All other residues were protonated according to each one's pK_a value at pH 7.4: all histidines were neutral and protonated only on the epsilon nitrogen, all lysines and arginines were protonated and positively charged, and glutamates and aspartates were deprotonated negatively charged.

To obtain its bound pose, PDBu was overlaid with the structurally-similar cocrystallized phorbol 13-acetate using ROCS.[14,15] The bryostatin 1 bound pose was found by first performing a conformational search using OMEGA,[16,17] docking these structures using FRED,[18,19,20] and using the highest scoring structure for further simulation. The orientation of this docked structure in the pocket is in agreement with what was first suggested by our group in 1988[21] and has since been used widely used to model bryostatin's bound pose in PKC. System preparation for prostratin and bryolog **3** was slightly different and is describe below. Ligand parameters for all

simulations were found using Antechamber and the general amber force field (GAFF)[22,23], and converted to gromacs format using acpype[24]. Angle parameters in the vicinity of the PDBu D-ring were not handled correctly by Antechamber, and were manually adjusted to give reasonable values (by default, Antechamber gave obviously implausible bond angles of 60° and 90° external to the cyclopropane, and were adjusted to the more reasonable 118° and 115°, respectively). Lipid parameters were derived from the Stockholm lipid (Slipid) parameters.[25] As palmitoyl oleoyl phosphatidylserine (POPS) was used in the experiments used to validate this study (see below), and these Slipid parameters have not been provided, the headgroup parameters of dioleoyl phosphatidylserine (DOPS) and the fatty acid parameters of palmitoyl oleoyl phosphatidylglycerol (POPG) were combined to yield the POPS parameters used. A membrane of 128 POPS monomers was assembled using Packmol.[26]

Simulation details

All simulations were performed using GROMACS 4.5.3.[27] Pulling simulations and equilibration were performed on a local cluster, and production simulations were run on the distributed computing platform Folding@home.[28] The AMBER99SB-ILDN[29] force field was used for proteins, GAFF[23] for ligands, Slipids[25] parameters for lipids, and TIP3P[30] for water. The system was constructed by placing the PKCδ C1b domain adjacent to the membrane (with PDBu or bryostatin bound, or without ligand) such that the binding site is nearest to the lipid headgroups and the N and C termini are furthest from it. This system was then solvated in an orthorhombic box where the dimensions were set by the x and y lengths of the bilayer, and such that water extended at least 1 nm from any heavy atoms in the z direction; 127 waters were replaced with Na^+ ions to neutralize the charges on both the protein and the phospholipids for a total of ~35000 atoms for each of the systems. All simulations were run with a 2 fs timestep. Structures were minimized using steepest descent with a cutoff of 1000 kJ/(mol x nm). The PDBu, bryostatin 1, and ligand-free systems were then equilibrated for 1 ns with the NVT ensemble, using the v-rescale thermostat[31] with a time constant of 0.1 ps at reference temperature 300K, followed by 100 ns of equilibration with the NPT ensemble using the Nosé-Hoover thermostat[32,33] at 300K, with a time constant of 0.5ps and three temperature groups (1: protein, zinc, and, ligand (if present), 2: lipids, and 3: waters and sodium). Pressure was controlled semi-isotropically using the Parrinello-Rahman barostat,[34] with a time constant of 10 ps, a reference pressure of 1 bar in both the x-y and z dimensions, and an isothermal compressibility of 4.5×10^{-5}/bar. All bonds were constrained using LINCS,[35] and long-range electrostatic interactions were treated using Particle-Mesh Ewald (PME),[36] with cubic interpolation and 0.12 nm grid spacing. Dispersion correction was turned off, as it was found to result in membranes with an unnaturally low area per lipid. Dispersion correction is best used in homogenous aqueous systems, not heterogeneous systems such as those including aqueous solvents and phospholipid membranes. The neighbor list was updated with a grid searching every 10 fs in all simulations. Short-range neighbor list, van der Waals, and electrostatic cutoffs were all set to 1.0 nm. Center-of-mass motions were removed independently for two groups (1: protein, zinc, lipids, and ligand (if present), 2: waters and sodium). Periodic boundary conditions were used for all simulations and randomized starting velocities were assigned from a Maxwell-Boltzmann distribution.

Upon equilibration, the PDBu, bryostatin 1, and ligand-free systems were pulled from solution into the membrane. The residues surrounding the binding pocket (amounting to approximately

one third of the residues of the protein), and, if present, the ligand, were the atoms pulled along the z axis from outside of the membrane to the interior, which consequently pulled the rest of the protein. In order not to induce deformation of the protein or the lipid bilayer, the systems were pulled slowly, over ~200 ns of simulation time. Approximately 100 snapshots from each system were taken from along the pulled coordinate, and each of these were equilibrated in the NPT ensemble as described above for 100 ns each. These equilibrated structures were then used as starting configurations for simulations on Folding@home. Each starting configuration corresponded to 40 clones, and they were simulated in the NPT ensemble according to the parameters described above for a total of 4000 trajectories per system and approximately 20 ns per trajectory. After this, a round of adaptive sampling was performed, in which snapshots from these simulations were used as new starting configurations such that all were evenly distributed across the observed free energy (see Supplementary Figure 10). In each system, approximately 1000 starting configurations, with 4 corresponding clones, were simulated, for 4000 additional trajectories per system. In total, the PDBu, bryostatin 1, and ligand-free systems each had approximately 400 µs aggregate simulation time and average trajectory length of 30 ns. For the prostratin simulations, the PDBu starting structures were directly modified, converting the C12 butyrate to a hydrogen and the C13 butyrate to an acetate. For the bryolog **3** simulations, the bryostatin starting structures were directly modified, converting the C8 *gem*-dimethyl to two hydrogens, the C9 OH to a hydrogen, and the C13 Z-enoate to be a simple exocyclic pyran olefin. For both prostratin and bryolog **3**, these changes had no impact upon ligand conformation. The resulting structures were minimized and simulated directly on Folding@home. The prostratin simulations had aggregate simulation time of 580 µs and average trajectory length of 115 ns. Bryolog **3** had aggregate simulation time of 200 µs and average trajectory length of 15 ns.

Data Analysis: Markov State Models

Each dataset was featurized using MSMBuilder 3.[37] Featurization is the process by which certain quantities particular to a system (such as the dihedral angles of the protein backbone) are aggregated from every relevant atom and every frame. This data (the system's "features") act as a way to describe the structure and dynamics of the system as a whole. Seven different featurizations were used to describe the system in this study. Three of these measured protein conformation in different ways (backbone dihedral angles, rmsd of protein when superposed with a reference structure, and distribution of reciprocal interatomic distances (DRID)[38] of all heavy atoms). Two measured the localization of water around the protein (the solvent shell featurizer[39] measures the local, instantaneous water density around all protein and ligand heavy atoms within a certain radius; in this case radii of 0.3 nm and 1.0 nm were used). Two measured the localization of lipid molecules around the protein and ligand (one used the same solvent shell featurizer except using lipids instead of waters, and the other used a weighted metric measuring the distance of every protein heavy atom to every lipid phosphorus atom[40]). These seven featurizations were combined such that each new one consisted of 0 or 1 characterization of the protein, water, and lipid systems. This yielded a total of 35 different featurizations for future analysis.

On each of these featurizations, we performed a tICA analysis.[41] We measured the four most slowly decorrelating linear combinations of these features, using a tICA lag time, *t*, between 0.5

ns and 5 ns and an L2 regularization strength gamma, *g*, between 10^{-1} and 10^{-10}. These tICs were then clustered into *k* states using the mini-batch k-means clustering method.[42] Using this clustering, Markov state models (MSMs) were built using lagtime of 4.5 ns. In order to determine optimal values for *t*, *g*, and *k* of the model, we sought to maximize the eigenvalues of our MSM, in accordance with the variational formulation of kinetics introduced by Nüske and Noé[43]. However, we were also cognizant that overfitting, arising for example due to an overly large value for *k* or overly small of a value for *g*, could pose a risk. For this reason, we selected these values by cross-validation using the variational GMRQ objective function described by McGibbon and Pande[44], which assesses how well the MSMs maximize a variational criterion evaluated on data that was held out during the fitting of the model. This optimization was managed by osprey (available at https://github.com/pandegroup/osprey), a tool for hyperparameter optimization of algorithms in machine learning.
Each iteration of the optimization involved building an MSM using a random subset of the data (the training set) and evaluating how slowly the first six eigenvectors of the MSM decorrelate when measured against the remaining subset of the data (the training set). Those that decorrelate the most slowly have the highest GMRQ scores and can be considered the "best" MSMs.
Projections shown in Supplementary Figure 11: The free energy histograms for the five top GMRQ-scoring MSMs are shown below for each system. show the top five GMRQ-scoring featurizations of PDBu, bryostatin 1, bryolog **3**, prostratin, and ligand-free systems, of which the histograms in Figure 3 in the main text are representative.

The implied timescale plots for all five different MSMs are shown below. The lagtime for all five MSMs was 9 frames (i.e., 4.5 ns). In all cases, the implied timescales flattened out well; qualitatively, this demonstrates positive results with regards to the Chapman Kolmogorov convergence metric. In the cases of bryolog **3** and bryostatin, there are discontinuities in the timescale plots (though they maintain local flatness). This is caused by a splitting of the eigenvalues and is not uncommon in MSMs. The GMRQ metric was developed in our lab in order to provide a more quantitative evaluation of MSM quality.

Data analysis: Free energy histograms

Projections of these systems onto free energy histograms were made by calculating several internal degrees of freedom of the protein. Because the PKCδ C1b domain is relatively cylindrical and has a well-defined "top" (near binding pocket) and "bottom" (near the C- and N-termini), the "depth" was calculated by taking the centroid of the coordinates four atoms near this "top" (the Cα atoms of M9, T12, L21, and V25), and computing the distance from this point to the plane of the center of the membrane (itself calculated by taking the mean of the z coordinate of every phosphorus atom in the membrane).

The angle with respect to the membrane was determined by calculating the angle between the x-y plane of the simulation box (which is approximately equal to the plane of the membrane itself) and a vector through the length of the peptide. This vector was defined as the line through the "top" point described above and a point near the "bottom", the centroid of the coordinates of four atoms (Cα for F3, G35, and N48, and the Zn^{2+} ion coordinated by H1, C30, C34, and C50). These atoms are relatively immobile, and the line through the top and bottom points acts as a good surrogate for the long axis of the peptide.

It is possible that conformational change in the protein might alter the location of the centroid points used to calculate angle and depth. This would distort the calculations of depth and angle without necessarily changing the protein's orientation in the membrane. Interestingly, we measured that very little conformational change to the C1b domain itself was observed across all ligands and orientations with the membrane, with the median and mean RMSD of backbone atoms per trajectory of only 0.2Å and 0.8Å, respectively. Thus, our assumption that the protein remains relatively rigid and cylindrical is well-founded.

When calculating the angle of the protein axis with the membrane, we are admittedly ignoring the rotation of the protein around this axis. Nevertheless, this is not a hugely significant flaw. There are several basic residues that reside along the length of one face of the PKCδ C1b domain (note the blue residues on the protein surface in Figure 2 in the main text), and these basic residues interact strongly with the anionic membrane. This precludes rotation around the protein's internal axis, as it would necessitate breaking these electrostatic contacts and incurring a large energetic penalty. Thus, the peptide possesses a relatively constant orientation of the basic residues towards the membrane, and rotation around its axis is not a significant factor.

The free energy histograms themselves were made by summing over all states of the MSM using the following equation: $W(x, y) = k_B T \ln[\sum_i^N \pi_i h_i(x, y)]$, *W(x, y)* is the function describing this free energy histogram, *N* is the number of states in the given MSM, *x* and *y* are the depth and angle of the protein, respectively, π_i is the probability of state *i*, and $h_i(x, y)$ is the normalized histogram of *x* and *y* from within state *i* specifically.

The relative free energies of the "shallow" and "deep" orientations for bryostatin 1 and bryolog **3** were calculated using the following equation: $\Delta\Delta G = -RT\ln\left(\frac{\sum \pi_{shallow}}{\sum \pi_{deep}}\right)$, where π_i are the populations of the "shallow" and "deep" regions of the bryostatin 1 and bryolog **3** free energy histograms.

Supplementary References

---

[1] Wang, Q. J. *et al.* Role of hydrophobic residues in the C1b domain of protein kinase C δ on ligand and phospholipid interactions. *J. Biol. Chem.* **276,** 19580-19587 (2001).
[2] Xu, R., Pawelczyk, T., Xia, T. H. & Brown, S. NMR structure of a protein kinase C-γ phorbol-binding domain and study of protein-lipid micelle interactions. *Biochemistry* **36,** 10709-10717 (1997).
[3] Ziemba, B. P. & Falke, J. J. Lateral diffusion of peripheral membrane proteins on supported lipid bilayers is controlled by the additive frictional drags of (1) bound lipids and (2) protein domains penetrating into the bilayer hydrocarbon core. *Chem. Phys. Lipids* **172-173,** 67-77 (2013).
[4] Shrake, A. & Rupley, J. A. Environment and exposure to solvent of protein atoms. Lysozyme and insulin. *J. Mol. Biol.* **79,** 351-364 (1973).
[5] Wender, P. A., Koehler, K. F., Sharkey, N. A., Dell'Aquila, M. L. & Blumberg, P. M. Analysis of the phorbol ester pharmacophore on protein kinase C as a guide to the rational design of new classes of analogs. *Proc. Natl. Acad. Sci. U.S.A.* **83,** 4214-4218 (1986).
[6] Pettit, G. R., Herald, C. L., Doubek, D. L. & Herald, D. L. Isolation and structure of bryostatin 1. *J. Am. Chem. Soc.* **104,** 6846-6848 (1982).
[7] Kamano, Y. *et al.* Conformational analysis of a marine antineoplastic macrolide, bryostatin 10. *Tetrahedron* **52,** 2369-2376 (1996).

[8] Wender, P. A., Loy, B. A. & Schrier, A. J. Translating nature's library: The bryostatins and function-oriented synthesis. *Isr. J. Chem.* **51,** 453-472.
[9] Wender, P. A. *et al.* Design, synthesis, and evaluation of potent bryostatin analogs that modulate PKC translocation selectivity. *Proc. Natl. Acad. Sci. USA* **108,** 6721-6726 (2001).
[10] Wender, P. A. & Verma, V. A. The design, synthesis, and evaluation of C7 diversified bryostatin analogs reveals a hot spot for PKC affinity. *Org. Lett.* **10,** 3331-3334 (2008).
[11] Zhang, G., Kazanietz, M., Blumberg, P. & Hurley, J. Crystal structure of the Cys2 activator-binding domain of protein kinase Cδ in complex with phorbol ester. *Cell* **81,** 917-924 (1995).
[12] Irie, K. *et al.* Molecular basis for protein kinase C isozyme-selective binding: The synthesis, folding, and phorbol ester binding of the cysteine-rich domains of all protein kinase C isozymes. *J. Am. Chem. Soc.* **120,** 9159-9167 (1998).
[13] Eswar, N. *et al.* UNIT 2.9 comparative protein structure modeling using MODELLER. *Curr. Protoc. Protein Sci* **50,** 2.9.1-2.9.31 (2007).
[14] ROCS 3.2.1.4: OpenEye Scientific Software, Santa Fe, NM. http://www.eyesopen.com.
[15] Hawkins, P. C. D., Skillman, A. G. & Nicholls, A. Comparison of Shape-Matching and Docking as Virtual Screening Tools. *J. Med. Chem.* **50,** 74 (2007).
[16] OMEGA 2.5.1.4: OpenEye Scientific Software, Santa Fe, NM. http://www.eyesopen.com.
[17] Hawkins, P. C. D., Skillman, A. G., Warren, G. L., Ellingson, B. A. & Stahl, M. T. Conformer generation with OMEGA: Algorithm and validation using high quality structures from the protein databank and Cambridge Structural Database. *J. Chem. Inf. Model.* **50,** 572-584 (2010).
[18] FRED 3.2.0.2 : OpenEye Scientific Software, Santa Fe, NM. http://www.eyesopen.com
[19] McGann, M. FRED pose prediction and virtual screening accuracy. *J. Chem. Inf. Model.* **51,** 578-596 (2011).
[20] McGann, M. FRED and HYBRID docking performance on standardized datasets. *J. Comput. Aided Mol. Des.* **26,** 897-906 (2012).
[21] Wender, P. A. *et al.* Modeling of the bryostatins to the phorbol ester pharmacophore on protein kinase C. *Proc. Natl. Acad. Sci. U.S.A.* **85,** 7197-7201 (1988).
[22] Wang, J., Wang, W., Kollman P. A. & Case, D. A. Automatic atom type and bond type perception in molecular mechanical calculations. *J. Mol. Graphics Modell.* **25,** 247-260 (2006).
[23] Wang, J., Wolf, R. M., Caldwell, J. W., Kollman, P. A. & Case, D. A. Development and testing of a general AMBER force field. *J. Comput. Chem.* **25,** 1157-1174 (2004).
[24] da Silva, A. S. & Vranken, W. ACPYPE - AnteChamber Python Parser interfacE. *BMC Research Notes* **5,** 367 (2012).
[25] Jämbeck, J. P. M., Lyubartsev, A. P. Another piece of the membrane puzzle: extending slipids further. *J. Chem. Theory Comput.* **9,** 774-784 (2013).
[26] Martínez, L., Andrade, R., Birgin, E.G. & Martínez, J. M. Packmol: A package for building initial configurations for molecular dynamics simulations. *J. Comput. Chem.* **30,** 2157-2164 (2009).
[27] Hess, B., Kutzner, C., van der Spoel, D. & Lindahl, E. Gromacs 4: algorithms for highly efficient, load-balanced, and scalable molecular simulation. *J. Chem. Theory. Comput.* **4,** 435-447 (2008).
[28] Shirts, M., Pande, V. S. Screen savers of the world unite! *Science* **298,** 1903-1904 (2000).
[29] Lindorff-Larsen, K. *et al.* Improved side-chain torsion potentials for the Amber ff99SB protein force field. *Proteins* **78,** 1950-1958 (2010).
[30] Jorgensen, W., Chandrasekhar, J., Madura, J., Impey, R. & Klein, M. Comparison of simple potential functions for simulating liquid water. *J. Chem Phys.* **79,** 926-935 (1983).
[31] Bussi, G., Donadio, D., Parrinello, M. Canonical sampling through velocity rescaling. *J. Chem. Phys.* **126,** 014101 (2007).
[32] Nosé, S. A unified formulation of the constant temperature molecular dynamics methods. *J. Chem. Phys.* **81,** 511-519 (1984).
[33] Hoover, W. G. Canonical dynamics: equilibrium phase-space distributions. *Phys. Rev. A: At. Mol. Opt. Phys.* **31,** 1695-1697 (1985).
[34] Parrinello, M., Rahman, A. Polymorphic transitions in single crystals: a new molecular dynamics method. *J. Appl. Phys.* **52,** 7182-7190 (1981).
[35] Hess, B., Bekker, H., Berendsen, H. J. C. & Fraaije, J. G. E. M. LINCS: a linear constraint solver for molecular simulations. *J. Comp Chem.* **18,** 1463-1472 (1997).
[36] Darden, T., York, D. & Pedersen, L. Particle mesh Ewald: an Nlog(N) method for Ewald sums in large systems. *J. Chem. Phys.* **98,** 10089-10092 (1993).

[37] Beauchamp, K.A. *et al.* MSMBuilder2: Modeling conformational dynamics on the picosecond to millisecond scale. *J. Chem. Theory Comput.* **7,** 3412-3419 (2011).
[38] Zhou, T. & Caflisch, A. Distribution of Reciprocal of Interatomic Distances: A Fast Structural Metric. *J. Chem. Theory Comput.* **8**, 2930-2937 (2012).
[39] Harrigan, M. P., Shukla, D. & Pande, V. S. Conserve water: A method for the analysis of solvent in molecular dynamics. *J. Chem. Theory Comput.* **11,** 1094-1101 (2015).
[40] Gu, C. *et al.* Building Markov state models with solvent dynamics. *BMC Bioinformatics* **14,** S8 (2013).
[41] Schwantes, C.R. & Pande, V.S. Improvements in Markov state model construction reveal many non-native interactions in the folding of NTL9. *J. Chem. Theory Comput.* **9,** 2000-2009 (2013).
[42] Sculley, D. Web-scale k-means clustering. In: *Proceedings of the 19th international conference on World wide web*, WWW. **10**, 1177–1178 (ACM, New York, NY, USA 2010).
[43] Nüske, F., Keller, B. G., Pérez-Hernández, G., Mey, A. S. J. S. & Noé, F. Variational approach to molecular kinetics. *J. Chem. Theory Comput.* **10,** 1739-1752 (2014).
[44] McGibbon, R. T. & Pande, V. S. Variational cross-validation of slow dynamical modes in molecular kinetics. *J. Chem. Phys.* **142,** 124105 (2015).
